# Supplementary material for: Tracing ancient solar cycles with tree rings and radiocarbon in the first millennium BCE
Source: Nat Commun. 2025 Jan 6;16:406. doi: 10.1038/s41467-024-55757-y (PMC11704142; doi:10.1038/s41467-024-55757-y)
Supplement: Supplementary file 1 — Supplementary Information [file 41467_2024_55757_MOESM1_ESM.pdf]

Supplementary Information for Tracing Ancient Solar Cycles with Tree Rings and Radiocarbon in the First Millennium BCE

*Nicolas Brehm[1,2], Charlotte L. Pearson[1], Marcus Christl[2], Alex Bayliss[3], Kurt Nicolussi[4], Thomas Pichler[4], David Brown[5] and *Lukas Wacker[2]

*corresponding Authors: nbrehm@arizona.edu; wacker@phys.ethz.ch

1. Dendrochronology

1.1 Dendrochronological dating of two bog oak wood samples from Swan Carr, Co. Durham, England (Q 4405 and Q 4416)

In July 1981, slices were collected from twenty bog oak (Quercus sp.) timbers from Swan Carr, Co. Durham, UK (54.63°N and 1.51°W).

The wet samples were prepared using a scalpel blade to remove the rough wood from the surface of the slice and to expose the tree-ring pattern. If the wood sample was soft or the ring pattern needed to be clarified, a razor blade was used. Finely ground chalk was rubbed onto the prepared surface to define the annual tree-ring boundaries more clearly for measurement. The ring-widths were measured to a precision of 1/50mm using a travelling stage from September 1981 to March 1982. Sometimes it was noted that the samples were likely to have come from the same tree.

The ring-width series of fifteen of these samples were included in the Swan Carr master chronology (Supplementary Table 1). The construction of the chronology followed the procedures described by Baillie (Baillie, Tree-ring dating and archaeology, 1982). Samples with the best correlation values were combined to form sub-site masters. These were then used to date other samples. The other dated samples were incorporated into a new sub-site master until a coherent master chronology was formed. The Swan Carr bog oak tree-ring chronology is the only English tree-ring chronology covering most of the first millennium BC. The individual samples that overlap with samples Q4405 and Q4416 were then compared with each other giving the correlation values presented in the *t*-value matrix (Supplementary Table 2). The correlation *t*-values are based on CROS84 (Munro, 1984).

Supplementary Table 1 Details of the cross-matched samples from the Swan Carr master chronology (no samples had sapwood or the heartwood-sapwood boundary present)

Sample	Rings	Absolute Dating
Q4402	150	1047–898 BCE
Q4403	443	1155–713 BCE
Q4404	173	864–692 BCE
Q4405	185	665–481 BCE
Q4407	240	922–683 BCE
Q4408	285	890–606 BCE
Q4410	335	1076–742 BCE
Q4411	250	675–426 BCE
Q4412	278	821–544 BCE
Q4413	273	692–420 BCE
Q4415	327	638–381 BCE

Q4416	143	569–427 BCE
Q4417	173	861–689 BCE
Q4418	261	1057–797 BCE
Q4420	305	830–526 BCE

*Supplementary Table 2 Truncated t-value matrix for ring-width series from the Swan Carr, Co. Durham chronology (- = no correlation value; nsm = non-significant match; nh = not highest match; * = significant match; ** = very significant match; *** = extremely significant match)*

	Q4420	Q4412	Q4413	Q4411	Q4405	Q4415	Q4416
Q4420	-	9.60***	2.40 nh	6.14***	4.29*	-	-
Q4412	-	-	1.94nh	6.02***	3.59 nsm	1.32 nh	-
Q4413	-	-	-	6.34***	4.14*	3.51 nsm	4.45*
Q4411	-	-	-	-	4.68*	5.80***	5.31**
Q4405	-	-	-	-	-	4.03 nsm	3.85 nsm
Q4415	-	-	-	-	-	-	2.63 nh

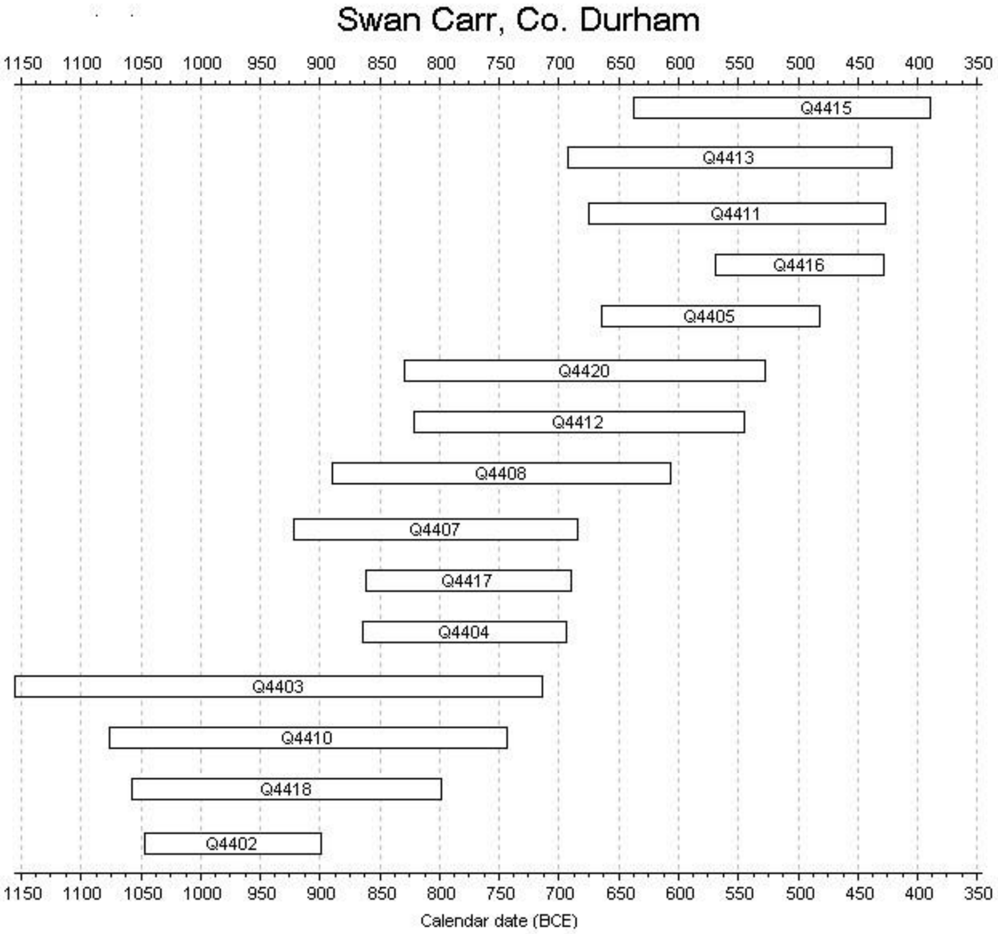

Supplementary Fig. 1 Bar diagram showing the relative positions of the dated samples from Swan Carr, Co. Durham

Timbers from Swan Carr were originally waterlogged, but subsequently have been allowed to dry out naturally. The remainder of the samples are stored in the Dendrochronology Laboratory, School of Natural and Built Environment, Queen's University, Belfast. Ring-width data for all the measured samples from Swan Carr, Co. Durham can be found at: http://chrono.qub.ac.uk>dendro_data>dendro

Two samples from the Swan Carr site chronology, Q4405 and Q4416, have been used to supply single-year samples for radiocarbon calibration. For the selected period, the trees with the widest annual growth rings were chosen, so that extracting the individual annual growth rings is as easy as possible. The samples to be used were cleaned with a razor blade and the annual growth rings marked. The annual growth rings were split from the bulk sample using a scalpel blade and the early wood growth was removed using a scalpel. This processing means that the wood samples provided only have the latewood part of the annual growth ring.

The original Swan Carr master chronology consists of 15 samples. It is 775 years in length and dates to 1155–381 BC (Brown, Munro, Baillie, & Pilcher, 1986). All positive correlation values over t = 4 are included in Supplementary Table 3. The correlation t-values are based on CROS84 (Munro, 1984).

*Supplementary Table 3 Correlation values[3] for the 775-year Swan Carr, Co. Durham master chronology with other site and regional chronologies (*** = extremely significant match; * = significant match; nsm = non-significant match)*

Site chronology	Start and end date of chronology	Correlation value with Swan Carr, Co. Durham
Göttingen, Germany	1368 BC–AD 120	8.45***
Garry Bog 2, Northern Ireland	947–229 BC	5.58***
Ballymacombs More, Northern Ireland	1303–949 BC	4.63*
Garry Bog 1, Northern Ireland	2516–975 BC	4.35 nsm

```
SWAN CARR
Q4405
185
  54  74  37  31  63  90  83  61  52  65
  32  14  27  94  27  44  55  57  86  88
  70  48  50  75  63  67  63  41  63  81
 107  96 102 122 107 121 184 100  79 104
 119 103 105 135 117  98 118 100 128 104
 123 104 111  83  96 132 108  86 121 115
 124  95  93  93  85  96  62  86  86 120
 106 125  65  90  96  96 126  99 150 116
 126 125 110 130 123 143 147 107 135  88
  90  85  89 105  69  59  82  89  95  87
  72  76 111  94  73  90  99  99 128 101
 104 104 103 102 125 104  80  75  86  62
  84 100  70  77  94  60  74  49  59  51
  80  33  57  47  46  22  33  31  18  18
  34  18  18  26  16  27  19  26  29  61
  59  52  43  74  72  58  65  36  47  42
  22  28  27  30  29  52  62  41  41  47
  77  50  77  95  74  89  60  43  40  26
  21  17  18  22  19
```

COMMENT – DMB Sept-1981

START DATE: 665BC
END DATE: 481BC

1.3 Dendrochronological dating of nine conifer samples from the Eastern Alps, Austria

Nine subfossil samples of three different tree species (larch / Larix decidua; spruce / Picea abies; cembran pine / Pinus cembra) originating from different sampling sites in the Alps (Tab. 1) and dating into the first millennium BCE were selected for the yearly resolved ^{14}C analyses. The main selection criterion was comparatively wide tree rings. The samples are stored in the Department of Geography, Universität Innsbruck, Innsbruck.

For all samples, tree-ring width measurements were made on several (partial) radii with a resolution of 1/1000 mm and subsequently averaged to establish sample mean series. These tree-ring width series were dated against and subsequently integrated into the Eastern Alpine Conifer Chronology (Nicolussi et al., 2009; 2015) and the later established and cross-dated Mitterberg chronology (Pichler et al., 2018) (Tab. 2). The tree-ring width series of the nine samples used are given below, the unit is always 1/1000 mm.

Supplementary Table 4 Overview of the sampling sites and species of the different dendrochronologically dated Alpine samples.

**Tree**	**Site**	**Coordinates**	**Species**
ahmo-63	Ahrntal, Moaralm	47°02'N / 12°05'E	cembran pine (*Pinus cembra*)
gli-27	Kaunertal, Ombrometer	46°53'N / 10°43'E	cembran pine (*Pinus cembra*)
gp-22	Kaunertal, Gepatschferner	46°52'N / 10°44'E	cembran pine (*Pinus cembra*)
hib-26	Defereggental, Hirschbichl	46°54'N / 12°15'E	larch (*Larix decidua*)
lfs-9	Langtaufers, Sandbichl	46°49'N / 10°42'E	cembran pine (*Pinus cembra*)
ssm-48	Zillertal, Schwarzensteinmoor	47°01'N / 11°49'E	cembran pine (*Pinus cembra*)
tah-29	Passeier, Timmeltal	46°54'N / 11°08'E	cembran pine (*Pinus cembra*)
vahu-1	Lesachtal, Wildsenderbach	46°44'N / 12°47'E	larch (*Larix decidua*)
vokb-79	Mitterberg, Vorderkeil	47°24'N / 13°09'E	spruce (*Picea abies*)

Supplementary Table 5 Crossdating statistics for the comparison of the utilized Alpine larch, spruce and cembran pine series with the supplemented (by spruce samples of the Mitterberg chronology) Eastern Alpine Conifer Chronology (Nicolussi, et al., 2009; Nicolussi, Weber, Patzelt, & Thurner, 2015; Pichler, et al., 2018). *Calculations were carried out by using the program WinTSAP. Overlap: no. of years; Glk.: Gleichläufigkeit; Sign. Glk.: pointer interval Gleichläufigkeit* (Eckstein & Bauch, 1969) *t-value$_{BP}$ and t-value$_{H}$: t-values after Baillie and Pilcher* (Baillie &

Pilcher, A simple cross-dating program for tree-ring research, 1973) *as well as Hollstein* (Hollstein, 1980) *dating: historical timescale, without year 0*.

Sample	Reference chronology	Overlap	Glk.	Sign. Glk.	t-value$_{BP}$	t-value$_{H}$	Dating
		[n]	(%]	[%]			[year BCE]
ahmo-63	EACCs	345	67	78	12.0	12.2	1120/776
gli-27	EACCs	155	72	86	10.6	9.7	490/336
gp-22	EACCs	236	68	86	8.5	7.4	952/717
hib-26	EACCs	151	73	95	9.6	11.6	1008/858
lfs-9	EACCs	126	64	100	4.3	4.6	510/385
ssm-48	EACCs	205	65	72	4.0	5.2	372/168
tah-29	EACCs	110	62	82	5.9	4.4	76/34 CE
vahu-1	EACCs	438	59	70	5.5	6.0	473/36
vokb-79	EACCs	154	68	100	6.4	7.1	538/385

HEADER:
KeyCode=ahmo-63
DateBegin=1120 BCE
DateEnd=776 BCE
DATA:

```
 2093  2086  1401  2275  2085  2051  2007  1163   747  1119
 1593  1226  1032   950   654   730   832  1487  1320  2024
 1401   867  1113  1426  1310  1143  1502  1159   998  1187
 1076  1296  1247   861  1234   919  1253  1299   896   979
  831  1077  1047   594   773   692   652   716  1015   899
 1013  1391  1205  1299  1261   922  1015  1004   859   895
  814   729   742   614   770   639   877   916   886   914
  594   632   754   800   903   895  1013   936   883   728
  856   620   645   612   728   638   590   583   785   718
  586   578   645   638   629   543   431   512   547   450
  399   484   583   588   540   528   593   610   697   667
  501   582   698   595   657   719   780   897   815   780
  757   820   700   693   697   628   617   604   589   558
  569   564   530   638   498   506   471   489   639   534
  563   578   527   526   654   654   622   612   735   541
  554   609   666   550   664   596   510   618   484   450
  615   659   639   546   586   465   634   709   589   592
  443   578   467   441   376   387   455   584   502   573
  496   505   502   484   460   404   326   344   329   312
  286   269   264   252   254   314   241   259   303   284
  297   258   148   180   200   178   194   196   239   182
  146   220   172   198   202   170   207   186   196   200
  174   216   199   268   262   233   195   229   231   226
  212   272   251   288   314   284   256   242   260   250
  259   238   211   280   262   285   246   254   195   196
  192   330   310   316   287   280   286   345   408   269
  302   262   323   337   290   262   239   248   272   275
  244   228   128   208   191   182   187   228   219   209
  258   242   286   298   227   324   350   306   326   324
  273   313   276   168   225   215   193   235   300   253
```

236 260 207 261 244 218 229 228 203 308
315 230 279 248 243 248 326 297 252 325
279 278 252 196 254 223 245 239 189 222
251 232 193 233 253 229 290 277 260 272
223 259 235 195 220

HEADER:
KeyCode=gli-27
DateBegin=490 BCE
DateEnd=336 BCE
DATA:
1519 2012 2456 1974 2017 1494 2913 2231 1943 2184
1462 1483 2058 1773 2074 1984 2100 1879 2505 2579
2093 1622 1863 1384 2020 1867 1411 1412 1978 1739
1417 1937 1525 1296 1222 1364 1112 1686 1847 1423
1947 2147 2065 2296 1977 1993 1821 2033 2490 2042
1716 2330 1998 2030 1817 2251 1985 1843 1834 1740
1447 2279 2043 1603 1667 828 1241 1451 1003 1828
1678 1876 1503 1960 1855 1542 1689 1325 1100 855
1152 1135 1216 1207 1192 911 1291 1053 1585 1597
1619 1351 1155 861 1267 1585 1502 1353 1387 1774
1467 1763 1497 1105 1295 1328 1120 1343 1064 1444
1694 1549 1527 1293 1475 1052 1012 1407 1306 1642
1261 1275 1149 1261 1250 824 1170 845 1353 1061
507 1110 980 923 695 880 1241 1071 1001 966
1121 1046 1023 1141 1284 1252 1838 1276 1507 1466
1534 1610 1073 1255 1427

HEADER:
KeyCode=gp-22
DateBegin=952 BCE
DateEnd=717 BCE
DATA:
1861 1493 1658 2053 2586 911 1276 1581 1631 1756
1395 1485 1723 1836 1545 1608 1421 1461 1540 1413
1505 1856 1620 1690 1846 1888 1645 1883 1886 1646
1721 1513 1505 1806 1588 1905 2095 2091 2030 1553
2046 1908 2355 2406 1945 2175 1970 1490 1038 1491
1213 1373 1301 1565 1493 1438 2018 1880 1588 1645
1218 1551 1642 1923 1603 1471 1626 1748 1116 1283
982 1256 1105 1083 735 768 625 791 673 836
820 1063 795 1003 1030 947 787 968 988 1171
1081 717 941 1091 716 1108 1027 868 1138 1035
917 973 1023 983 677 751 970 953 1078 857
657 815 721 610 770 691 531 625 871 922
927 973 918 1007 982 817 976 1186 721 805
790 880 935 993 967 992 736 865 890 942
863 996 990 1031 953 821 701 632 845 807
644 615 673 705 703 710 849 812 1059 828
710 638 735 633 625 592 640 644 693 774
600 522 495 498 471 509 506 431 340 359
286 345 273 230 254 260 290 224 280 320
294 281 291 238 344 292 328 321 262 285
325 308 302 338 380 400 320 300 303 297
273 323 246 316 378 310 296 241 268 316
296 323 353 275 287 235 242 295 267 325
320 342 242 202 155 100

HEADER:
KeyCode=hib-26
DateBegin=1008 BCE
DateEnd=858 BCE
DATA:
1864 2485 2070 1978 1848 2210 1894 2273 1859 1527
1803 1742 1839 1762 1455 1606 1500 1350 1567 1639
1444 1290 1148 1317 1369 1123 1343 1436 1433 1415
1269 1474 1369 1483 1281 1264 1412 1120 1095 1237
1384 1157 1256 1513 1450 1536 1727 1811 1656 1653
1411 1286 1506 1250 1451 1270 1419 1304 734 1258
893 1063 1033 1068 975 1274 1400 1598 1439 1446
1298 1329 1202 1062 776 920 799 1054 1134 1041
1008 1003 715 1132 902 922 917 717 819 759
428 767 867 795 821 577 970 772 516 874
728 656 735 336 509 593 635 718 658 807
701 693 882 816 926 909 536 559 563 900
581 689 653 661 285 417 597 572 486 516
434 498 428 485 550 567 348 301 273 456
400 335 352 387 316 369 313 334 291 170
209

HEADER:
KeyCode=lfs-9
DateBegin=510 BCE
DateEnd=385 BCE
DATA:
830 970 1040 1193 1263 1535 1546 802 704 893
1352 1012 1162 1307 951 1311 1244 1048 1347 691
689 1041 1115 688 660 587 1121 1017 1319 1211
1340 1731 1818 1681 1474 1673 1912 1968 2180 2075
2107 2652 2458 1796 2404 1850 1502 1329 1351 1191
1067 1116 1198 871 916 1339 1091 1428 1504 1397
1342 1709 1931 1947 1888 1767 1971 1736 2164 2189
2078 2235 2032 1990 1781 1695 1684 1420 1387 1495
1015 1501 1527 1817 2043 1347 1863 1673 1264 1994
2011 2125 1549 2154 1978 2130 1934 2048 1519 1306
1642 1477 1819 1470 1346 1091 1324 1544 1718 1625
1787 1525 1561 1172 1758 1614 1558 1359 1414 1734
1524 1827 1593 960 877 827

HEADER:
KeyCode=ssm-48
DateBegin=372 BCE
DateEnd=168 BCE
DATA:
668 662 601 524 547 441 403 337 485 492
676 513 397 281 283 321 137 155 182 330
639 907 1392 1548 1745 1619 1710 1025 1541 1271
1525 742 699 1187 1182 1217 1448 1643 1930 1839
1612 1670 1932 1652 1277 1084 1172 986 1158 1247
1430 1510 1223 979 1079 712 1150 1295 1422 1497
1880 1995 2676 2091 2243 2364 2276 2402 2104 1816
1639 1839 1668 1296 1211 1320 1851 1004 712 1033
916 757 903 848 815 565 859 746 688 814
749 691 984 1099 833 849 920 828 870 948
971 1006 1112 1152 1679 1382 1639 1336 1760 1660
1975 1726 2008 1434 1166 1065 847 774 1311 1784

1301 1505 1703 1514 1072 920 707 871 970 1032
1015 1164 1105 860 1018 701 727 796 871 1023
1262 1402 1367 1555 1526 1205 1025 788 652 707
742 861 970 1002 914 969 823 904 864 1148
920 897 1100 839 1219 1155 997 748 800 981
906 1176 1116 1190 993 639 943 956 766 1068
1045 1577 1574 1530 1040 955 1209 1223 2105 1983
1476 1302 1356 796 763 1177 1407 1813 1862 2575
1873 1517 1302 1424 850

HEADER:
KeyCode=tah-29
DateBegin=76 BCE
DateEnd=34 CE
DATA:
1272 1273 1045 1043 737 837 677 633 672 435
724 567 705 544 550 768 674 785 850 961
1002 773 831 846 726 901 915 799 1095 1141
917 966 1088 965 1146 1367 928 710 641 423
622 592 776 796 739 816 757 910 891 1353
1155 1216 1121 1022 1015 1122 1254 1046 1087 905
930 781 1020 780 1053 1118 1035 957 867 580
863 1014 1016 989 943 931 730 490 429 444
503 576 737 624 635 655 676 694 730 813
697 583 806 851 715 590 580 583 564 461
413 433 477 430 418 370 346 496 700 689

HEADER:
KeyCode=vahu-1
DateBegin=473 BCE
DateEnd=36 BCE
DATA:
768 627 587 398 411 261 245 127 343 131
187 490 240 243 216 302 320 140 112 176
150 168 135 158 232 250 233 127 146 271
226 381 542 412 496 645 721 1071 1011 763
716 583 500 338 326 395 331 366 358 220
448 420 283 242 173 196 231 373 318 345
362 268 270 417 458 855 556 385 445 553
592 536 405 298 381 385 495 522 548 537
552 482 548 745 751 908 891 701 720 722
686 556 670 791 855 670 637 583 435 655
615 668 643 835 625 528 757 652 720 605
762 723 745 466 531 515 533 552 483 623
618 585 685 622 742 645 706 812 773 851
747 721 1110 850 823 857 773 921 1093 848
936 791 963 820 665 508 663 496 575 660
756 517 477 513 793 486 792 978 1148 856
1086 1305 1247 1076 1137 1206 980 983 912 1116
767 827 846 801 796 690 846 866 817 685
841 713 1080 836 732 551 896 966 840 922
1130 1080 1237 961 771 752 1067 823 961 732
1217 875 611 435 773 1040 878 1028 776 655
645 888 788 1020 885 852 985 787 311 827
660 966 1033 702 941 993 1003 768 803 857
906 843 910 783 816 642 736 722 682 743
986 961 1108 827 981 892 688 763 926 859
875 934 722 582 886 861 638 506 478 617

511 544 383 676 765 569 628 455 527 317
482 588 566 496 601 522 538 762 908 700
949 886 769 654 776 942 829 580 472 611
723 566 726 408 527 626 723 478 617 743
881 883 973 750 704 609 891 1024 1098 1205
929 838 641 821 807 1083 1584 1118 880 837
950 901 790 878 780 811 739 985 812 635
665 578 538 527 661 724 509 534 575 700
772 911 760 782 420 454 412 776 752 812
994 1062 822 998 1042 1286 837 588 473 615
434 518 698 748 750 566 747 480 599 616
592 743 757 801 447 742 551 422 724 500
656 596 577 594 731 947 549 606 539 640
686 719 822 686 671 623 580 668 723 824
903 863 791 550 367 541 442 694 1046 895
506 451 729 617 572 426 292 236 649 828
322 571 627 408 514 508 563 460 265 333
309 465 424 447 315 307 585 560

HEADER:
KeyCode=vokb-79
DateBegin=538 BCE
DateEnd=385 BCE
DATA:
2056 2204 2607 2134 2386 1457 2126 1702 1777 1271
1158 1119 760 788 881 840 989 949 878 1028
1102 1051 1110 1003 686 1129 1036 1223 900 827
930 819 876 775 953 1134 814 1010 951 643
953 856 762 834 857 804 926 1045 918 779
1071 1043 707 828 867 911 1034 934 916 908
890 831 819 813 796 612 699 611 710 751
742 843 900 846 600 644 531 725 674 758
659 849 893 912 794 808 635 835 596 611
675 429 550 759 574 625 613 510 419 517
477 498 532 461 448 441 497 421 402 488
599 624 524 337 523 635 418 387 454 419
418 400 394 450 596 475 425 459 452 409
336 292 318 206 192 210 197 262 239 207
224 212 173 146 117 105 125 130 124 124
154 161 171 132

2. Detrending of the 664 BCE event

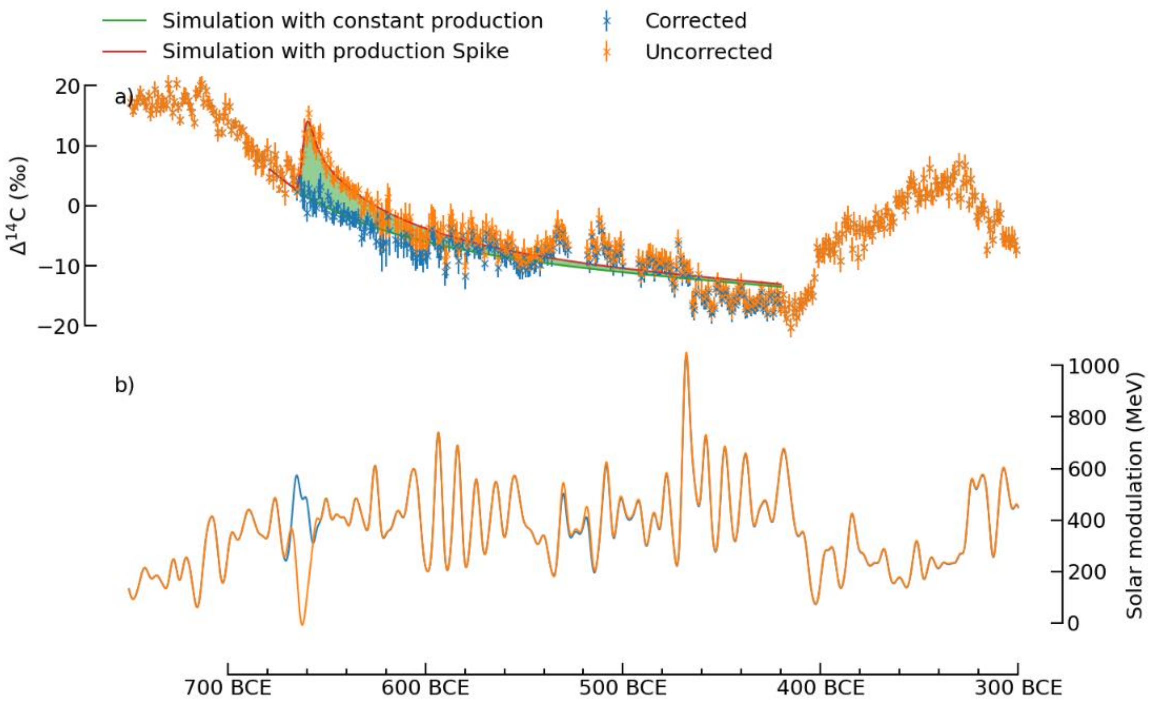

Supplementary Fig. 2 Illustration of the data detrending around the 664 BCE event. a) Uncorrected $\Delta^{14}C$ record around the 664 BCE event (orange) with simulated $\Delta^{14}C$ with a constant ^{14}C production (green) and a production spike (red) The error bars indicate the 2-σ uncertainty range. The corrected $\Delta^{14}C$ record (blue) was determined by subtracting the difference of the simulations from the uncorrected data. b) Resulting solar modulation parameter Φ by using the uncorrected (orange) and corrected (blue) $\Delta^{14}C$ record.

3. Quality control

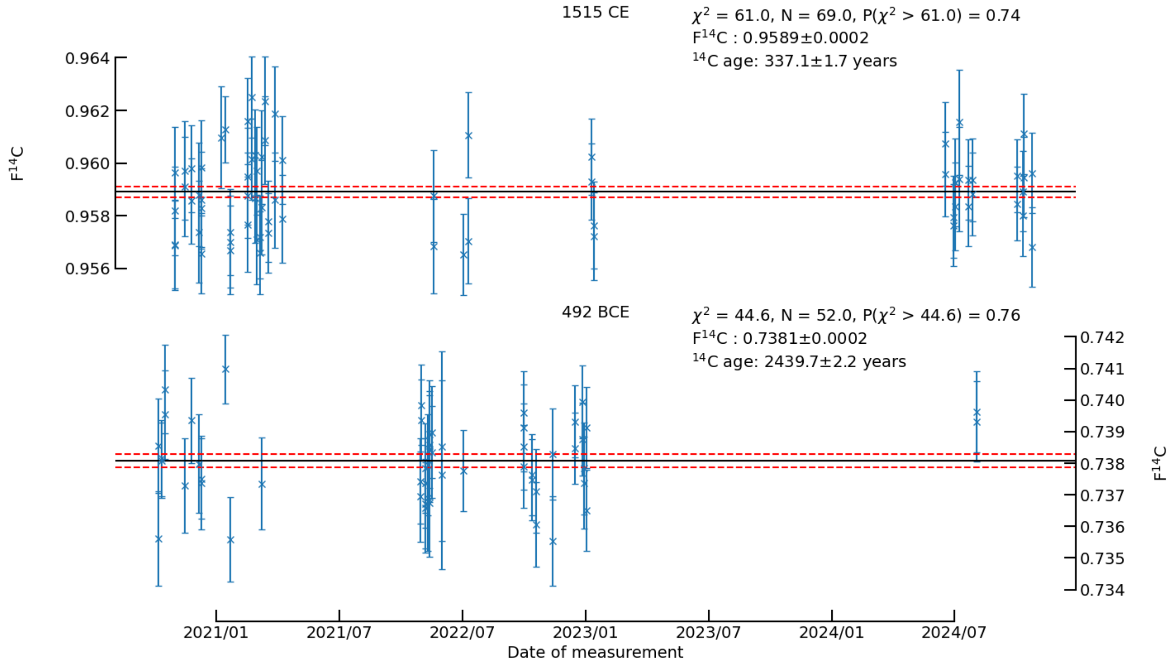

Supplementary Fig. 3 Measurement results of the two reference materials. The black lines indicate the mean value of the measurements and the red lines indicate the error of the mean. The error bars indicate the 2-σ uncertainty range.

4. Radiocarbon Box model

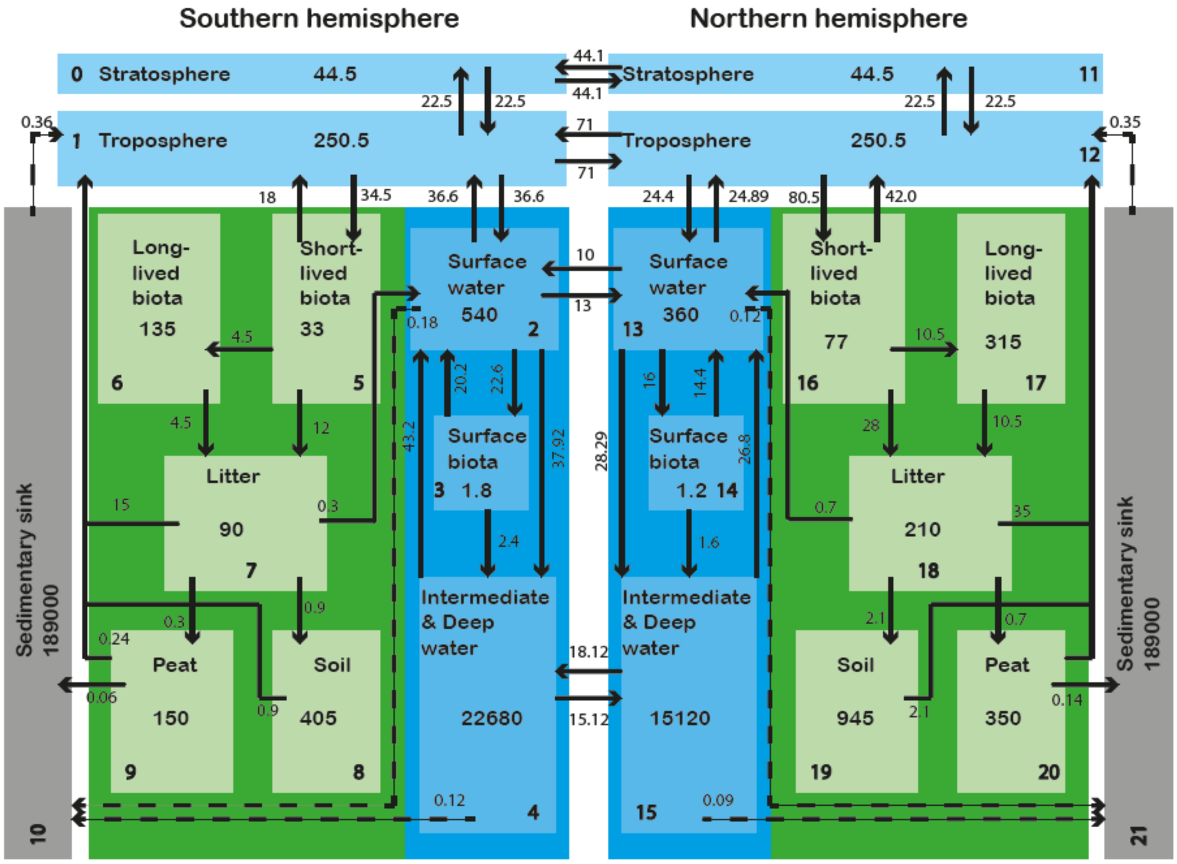

Supplementary Fig. 4 Depiction of carbon cycle box model. The fluxes are given in Gt/yr and the ^{12}C box contents are given in Gt of carbon.

5. Reconstruction of solar modulation

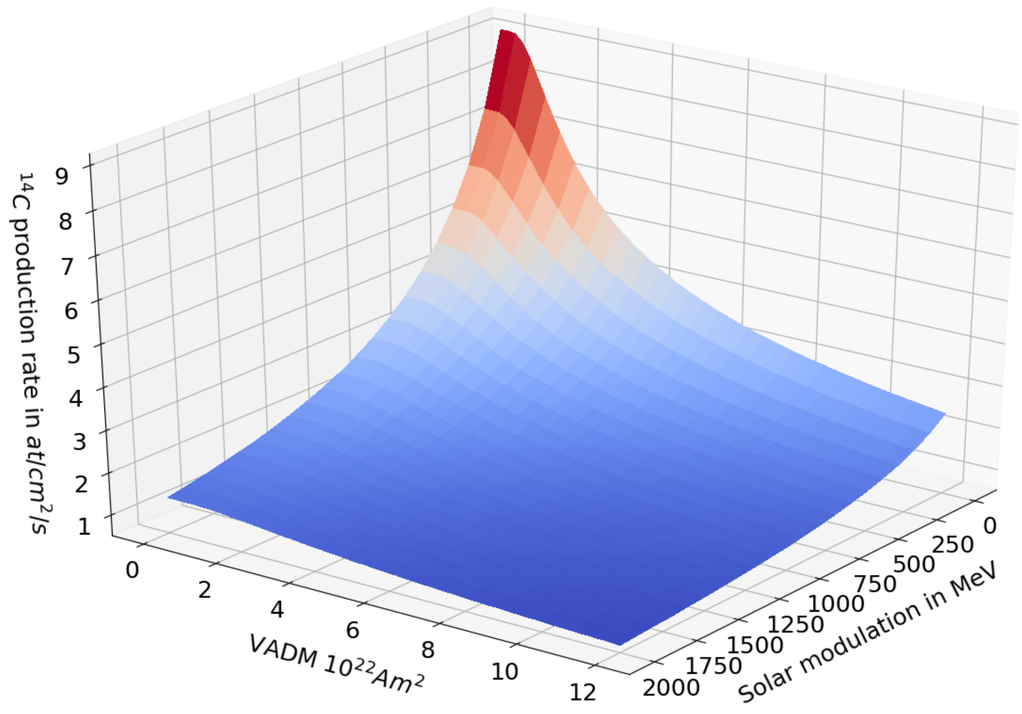

Supplementary Fig. 5 ^{14}C production rate dependence on earth's virual axis dipole moment and solar modulation parameter (Herbst, Muscheler, & Heber, 2017).

5. Radiocarbon measurement results

Lab Nr.	Labcode	Tree	Year BP	^{14}C Age ± σ (yr BP)	$F^{14}C$ ± σ
118906.1.1	ETH	Q4405	2478	2469 ± 16	0.7354 ± 0.0014
118906.1.2	ETH	Q4405	2478	2452 ± 15	0.7370 ± 0.0014
118907.1.1	ETH	Q4405	2479	2453 ± 15	0.7369 ± 0.0014
118908.1.1	ETH	Q4405	2480	2449 ± 15	0.7372 ± 0.0014
118909.1.1	ETH	Q4405	2481	2446 ± 15	0.7375 ± 0.0014
118909.1.2	ETH	Q4405	2481	2434 ± 15	0.7386 ± 0.0014
118910.1.1	ETH	Q4405	2482	2453 ± 15	0.7369 ± 0.0014
118911.1.1	ETH	Q4405	2483	2434 ± 15	0.7386 ± 0.0014
118912.1.1	ETH	Q4405	2484	2449 ± 16	0.7372 ± 0.0014
118912.1.2	ETH	Q4405	2484	2440 ± 15	0.7380 ± 0.0014
118913.1.1	ETH	Q4405	2485	2462 ± 15	0.7360 ± 0.0014
118914.1.1	ETH	Q4405	2486	2474 ± 15	0.7349 ± 0.0014
118915.1.1	ETH	Q4405	2487	2460 ± 16	0.7362 ± 0.0014
118915.1.2	ETH	Q4405	2487	2475 ± 15	0.7349 ± 0.0014
118916.1.1	ETH	Q4405	2488	2474 ± 15	0.7349 ± 0.0014
118917.1.1	ETH	Q4405	2489	2467 ± 15	0.7356 ± 0.0014
118918.1.1	ETH	Q4405	2490	2469 ± 15	0.7354 ± 0.0014
118918.1.2	ETH	Q4405	2490	2488 ± 15	0.7337 ± 0.0014
118919.1.1	ETH	Q4405	2491	2481 ± 16	0.7343 ± 0.0014
118920.1.1	ETH	Q4405	2492	2493 ± 15	0.7332 ± 0.0014
118921.1.1	ETH	Q4405	2493	2477 ± 15	0.7347 ± 0.0014
118921.1.2	ETH	Q4405	2493	2468 ± 15	0.7355 ± 0.0014
118922.1.1	ETH	Q4405	2494	2489 ± 16	0.7336 ± 0.0014
118923.1.1	ETH	Q4405	2495	2493 ± 15	0.7332 ± 0.0014
118924.1.1	ETH	Q4405	2496	2479 ± 15	0.7345 ± 0.0014
118924.1.2	ETH	Q4405	2496	2506 ± 16	0.7320 ± 0.0014
118925.1.1	ETH	Q4405	2497	2498 ± 15	0.7327 ± 0.0014

118926.1.1	ETH	Q4405	2498	2500 ± 16	0.7326 ± 0.0014
118927.1.1	ETH	Q4405	2499	2506 ± 16	0.7320 ± 0.0014
118927.1.2	ETH	Q4405	2499	2505 ± 15	0.7321 ± 0.0014
118928.1.1	ETH	Q4405	2500	2489 ± 16	0.7335 ± 0.0014
118929.1.1	ETH	Q4405	2501	2504 ± 15	0.7322 ± 0.0014
118930.1.1	ETH	Q4405	2502	2521 ± 16	0.7307 ± 0.0014
118930.1.2	ETH	Q4405	2502	2499 ± 15	0.7327 ± 0.0014
118931.1.1	ETH	Q4405	2503	2502 ± 15	0.7324 ± 0.0014
118932.1.1	ETH	Q4405	2504	2502 ± 14	0.7324 ± 0.0013
118933.1.1	ETH	Q4405	2505	2496 ± 14	0.7329 ± 0.0013
118933.1.2	ETH	Q4405	2505	2505 ± 15	0.7321 ± 0.0014
118934.1.1	ETH	Q4405	2506	2483 ± 14	0.7341 ± 0.0013
118935.1.1	ETH	Q4405	2507	2491 ± 14	0.7333 ± 0.0013
118936.1.1	ETH	Q4405	2508	2474 ± 14	0.7349 ± 0.0013
118936.1.2	ETH	Q4405	2508	2485 ± 15	0.7339 ± 0.0014
118937.1.1	ETH	Q4405	2509	2498 ± 14	0.7328 ± 0.0013
118938.1.1	ETH	Q4405	2510	2497 ± 14	0.7329 ± 0.0013
118939.1.1	ETH	Q4405	2511	2494 ± 14	0.7331 ± 0.0013
118939.1.2	ETH	Q4405	2511	2498 ± 15	0.7327 ± 0.0014
118940.1.1	ETH	Q4405	2512	2489 ± 14	0.7335 ± 0.0013
118941.1.1	ETH	Q4405	2513	2514 ± 14	0.7313 ± 0.0013
118942.1.1	ETH	Q4405	2514	2460 ± 14	0.7362 ± 0.0013
118942.1.2	ETH	Q4405	2514	2501 ± 15	0.7324 ± 0.0014
118943.1.1	ETH	Q4405	2515	2491 ± 14	0.7334 ± 0.0013
118944.1.1	ETH	Q4405	2516	2474 ± 14	0.7349 ± 0.0013
118945.1.1	ETH	Q4405	2517	2509 ± 14	0.7317 ± 0.0013
118945.1.2	ETH	Q4405	2517	2457 ± 15	0.7364 ± 0.0014
118946.1.1	ETH	Q4405	2518	2489 ± 14	0.7336 ± 0.0013
118947.1.1	ETH	Q4405	2519	2472 ± 14	0.7351 ± 0.0013
118948.1.1	ETH	Q4405	2520	2526 ± 14	0.7302 ± 0.0013
118948.1.2	ETH	Q4405	2520	2499 ± 15	0.7326 ± 0.0014
118949.1.1	ETH	Q4405	2521	2483 ± 14	0.7341 ± 0.0013
118950.1.1	ETH	Q4405	2522	2497 ± 14	0.7328 ± 0.0013
118951.1.1	ETH	Q4405	2523	2511 ± 14	0.7315 ± 0.0013
118951.1.2	ETH	Q4405	2523	2488 ± 15	0.7337 ± 0.0014
118952.1.1	ETH	Q4405	2524	2492 ± 14	0.7333 ± 0.0013
118953.1.1	ETH	Q4405	2525	2498 ± 14	0.7327 ± 0.0013
118954.1.1	ETH	Q4405	2526	2479 ± 14	0.7345 ± 0.0013
118954.1.2	ETH	Q4405	2526	2500 ± 33	0.7325 ± 0.0030
118955.1.1	ETH	Q4405	2527	2475 ± 14	0.7348 ± 0.0013
118956.1.1	ETH	Q4405	2528	2493 ± 18	0.7332 ± 0.0017
118957.1.1	ETH	Q4405	2529	2509 ± 18	0.7317 ± 0.0016
118957.1.2	ETH	Q4405	2529	2493 ± 33	0.7332 ± 0.0030
118958.1.1	ETH	Q4405	2530	2539 ± 18	0.7290 ± 0.0017
118959.1.1	ETH	Q4405	2531	2486 ± 18	0.7339 ± 0.0016
118960.1.1	ETH	Q4405	2532	2501 ± 18	0.7325 ± 0.0016
118960.1.2	ETH	Q4405	2532	2498 ± 33	0.7327 ± 0.0030
118961.1.1	ETH	Q4405	2533	2521 ± 18	0.7306 ± 0.0016
118962.1.1	ETH	Q4405	2534	2500 ± 18	0.7325 ± 0.0016
118963.1.1	ETH	Q4405	2535	2511 ± 18	0.7316 ± 0.0016
118963.1.2	ETH	Q4405	2535	2476 ± 33	0.7347 ± 0.0030
118964.1.1	ETH	Q4405	2536	2486 ± 18	0.7338 ± 0.0016
118965.1.1	ETH	Q4405	2537	2480 ± 18	0.7344 ± 0.0016
118966.1.1	ETH	Q4405	2538	2502 ± 19	0.7324 ± 0.0018
118966.1.2	ETH	Q4405	2538	2481 ± 33	0.7343 ± 0.0030
118967.1.1	ETH	Q4405	2539	2531 ± 18	0.7297 ± 0.0017
118968.1.1	ETH	Q4405	2540	2542 ± 18	0.7288 ± 0.0017
118969.1.1	ETH	Q4405	2541	2492 ± 18	0.7333 ± 0.0016
118969.1.2	ETH	Q4405	2541	2517 ± 33	0.7310 ± 0.0030

118970.1.1	ETH	Q4405	2542	2509 ± 18	0.7317 ± 0.0017
118971.1.1	ETH	Q4405	2543	2519 ± 19	0.7308 ± 0.0017
118972.1.1	ETH	Q4405	2544	2513 ± 18	0.7314 ± 0.0016
118972.1.2	ETH	Q4405	2544	2498 ± 33	0.7327 ± 0.0030
118973.1.1	ETH	Q4405	2545	2497 ± 19	0.7329 ± 0.0017
118974.1.1	ETH	Q4405	2546	2493 ± 18	0.7332 ± 0.0016
118975.1.1	ETH	Q4405	2547	2489 ± 18	0.7336 ± 0.0016
118975.1.2	ETH	Q4405	2547	2486 ± 33	0.7339 ± 0.0030
118976.1.1	ETH	Q4405	2548	2505 ± 18	0.7321 ± 0.0016
118977.1.1	ETH	Q4405	2549	2526 ± 18	0.7302 ± 0.0017
118978.1.1	ETH	Q4405	2550	2520 ± 19	0.7308 ± 0.0017
118978.1.2	ETH	Q4405	2550	2530 ± 33	0.7298 ± 0.0030
118979.1.1	ETH	Q4405	2551	2532 ± 18	0.7296 ± 0.0016
118980.1.1	ETH	Q4405	2552	2523 ± 18	0.7305 ± 0.0017
118981.1.1	ETH	Q4405	2553	2525 ± 18	0.7303 ± 0.0017
118981.1.2	ETH	Q4405	2553	2529 ± 33	0.7299 ± 0.0030
118982.1.1	ETH	Q4405	2554	2529 ± 19	0.7299 ± 0.0017
118983.1.1	ETH	Q4405	2555	2532 ± 19	0.7296 ± 0.0017
118984.1.1	ETH	Q4405	2556	2532 ± 19	0.7296 ± 0.0017
118984.1.2	ETH	Q4405	2556	2515 ± 33	0.7312 ± 0.0030
118985.1.1	ETH	Q4405	2557	2520 ± 19	0.7308 ± 0.0017
118986.1.1	ETH	Q4405	2558	2501 ± 19	0.7325 ± 0.0017
118987.1.1	ETH	Q4405	2559	2520 ± 19	0.7308 ± 0.0017
118987.1.2	ETH	Q4405	2559	2513 ± 33	0.7314 ± 0.0030
118988.1.1	ETH	Q4405	2560	2502 ± 19	0.7324 ± 0.0017
118989.1.1	ETH	Q4405	2561	2519 ± 19	0.7309 ± 0.0017
118990.1.1	ETH	Q4405	2562	2503 ± 19	0.7323 ± 0.0017
118990.1.2	ETH	Q4405	2562	2526 ± 33	0.7302 ± 0.0030
118992.1.1	ETH	Q4405	2564	2519 ± 19	0.7308 ± 0.0017
118993.1.1	ETH	Q4405	2565	2517 ± 19	0.7310 ± 0.0017
118993.1.2	ETH	Q4405	2565	2506 ± 33	0.7320 ± 0.0030
118996.1.2	ETH	Q4405	2568	2504 ± 33	0.7322 ± 0.0030
118997.1.1	ETH	Q4405	2569	2485 ± 18	0.7339 ± 0.0017
118998.1.1	ETH	Q4405	2570	2520 ± 33	0.7307 ± 0.0030
118999.1.1	ETH	Q4405	2571	2522 ± 16	0.7305 ± 0.0015
118999.1.2	ETH	Q4405	2571	2514 ± 33	0.7313 ± 0.0030
119000.1.1	ETH	Q4405	2572	2527 ± 33	0.7301 ± 0.0030
119001.1.1	ETH	Q4405	2573	2523 ± 16	0.7304 ± 0.0015
119002.1.1	ETH	Q4405	2574	2495 ± 16	0.7330 ± 0.0015
119002.1.2	ETH	Q4405	2574	2498 ± 33	0.7327 ± 0.0030
119003.1.1	ETH	Q4405	2575	2528 ± 17	0.7300 ± 0.0015
119004.1.1	ETH	Q4405	2576	2482 ± 16	0.7342 ± 0.0015
119005.1.1	ETH	Q4405	2577	2492 ± 16	0.7333 ± 0.0015
119005.1.2	ETH	Q4405	2577	2522 ± 33	0.7306 ± 0.0030
119006.1.1	ETH	Q4405	2578	2521 ± 16	0.7307 ± 0.0015
119007.1.1	ETH	Q4405	2579	2494 ± 16	0.7331 ± 0.0015
119008.1.1	ETH	Q4405	2580	2503 ± 16	0.7323 ± 0.0015
119008.1.2	ETH	Q4405	2580	2499 ± 33	0.7327 ± 0.0030
119009.1.1	ETH	Q4405	2581	2515 ± 16	0.7311 ± 0.0015
119010.1.1	ETH	Q4405	2582	2521 ± 16	0.7307 ± 0.0015
119011.1.1	ETH	Q4405	2583	2519 ± 16	0.7308 ± 0.0014
119011.1.2	ETH	Q4405	2583	2502 ± 33	0.7324 ± 0.0030
119012.1.1	ETH	Q4405	2584	2519 ± 16	0.7309 ± 0.0014
119013.1.1	ETH	Q4405	2585	2503 ± 16	0.7323 ± 0.0014
119014.1.1	ETH	Q4405	2586	2506 ± 16	0.7321 ± 0.0015
119014.1.2	ETH	Q4405	2586	2496 ± 33	0.7329 ± 0.0030
119015.1.1	ETH	Q4405	2587	2505 ± 16	0.7321 ± 0.0014
119016.1.1	ETH	Q4405	2588	2496 ± 16	0.7329 ± 0.0015
119017.1.1	ETH	Q4405	2589	2497 ± 16	0.7328 ± 0.0015

119017.1.2	ETH	Q4405	2589	2498 ± 33	0.7327 ± 0.0030
119018.1.1	ETH	Q4405	2590	2478 ± 16	0.7345 ± 0.0015
119019.1.1	ETH	Q4405	2591	2479 ± 16	0.7345 ± 0.0015
119020.1.1	ETH	Q4405	2592	2500 ± 16	0.7326 ± 0.0014
119020.1.2	ETH	Q4405	2592	2475 ± 33	0.7348 ± 0.0030
119021.1.1	ETH	Q4405	2593	2499 ± 16	0.7327 ± 0.0014
119022.1.1	ETH	Q4405	2594	2488 ± 16	0.7336 ± 0.0014
119023.1.1	ETH	Q4405	2595	2493 ± 16	0.7331 ± 0.0014
119023.1.2	ETH	Q4405	2595	2469 ± 33	0.7354 ± 0.0030
119024.1.1	ETH	Q4405	2596	2513 ± 16	0.7313 ± 0.0014
119025.1.1	ETH	Q4405	2597	2501 ± 16	0.7324 ± 0.0014
119026.1.1	ETH	Q4405	2598	2457 ± 15	0.7365 ± 0.0014
119026.1.2	ETH	Q4405	2598	2481 ± 15	0.7343 ± 0.0014
119027.1.1	ETH	Q4405	2599	2466 ± 15	0.7357 ± 0.0014
119028.1.1	ETH	Q4405	2600	2491 ± 15	0.7334 ± 0.0014
119029.1.1	ETH	Q4405	2601	2478 ± 15	0.7346 ± 0.0014
119029.1.2	ETH	Q4405	2601	2463 ± 15	0.7359 ± 0.0014
119030.1.1	ETH	Q4405	2602	2458 ± 15	0.7364 ± 0.0014
140907.1.1	ETH	Q4405	2478	2426 ± 14	0.7393 ± 0.0013
140908.1.1	ETH	Q4405	2477	2467 ± 14	0.7356 ± 0.0013
140909.1.1	ETH	Q4405	2476	2461 ± 14	0.7362 ± 0.0013
140910.1.1	ETH	Q4405	2475	2448 ± 14	0.7373 ± 0.0013
140911.1.1	ETH	Q4405	2474	2444 ± 14	0.7377 ± 0.0013
140912.1.1	ETH	Q4405	2473	2458 ± 14	0.7364 ± 0.0013
140913.1.1	ETH	Q4405	2472	2449 ± 15	0.7373 ± 0.0014
140914.1.1	ETH	Q4405	2471	2470 ± 15	0.7353 ± 0.0013
140915.1.1	ETH	Q4405	2470	2452 ± 15	0.7370 ± 0.0013
140916.1.1	ETH	Q4405	2469	2443 ± 14	0.7377 ± 0.0013
140917.1.1	ETH	Q4405	2468	2462 ± 14	0.7360 ± 0.0013
124145.1.1	ETH	Q4416	2468	2458 ± 15	0.7364 ± 0.0013
124146.1.1	ETH	Q4416	2467	2467 ± 15	0.7356 ± 0.0013
124147.1.1	ETH	Q4416	2466	2431 ± 15	0.7389 ± 0.0014
124148.1.1	ETH	Q4416	2465	2443 ± 15	0.7378 ± 0.0013
124149.1.1	ETH	Q4416	2464	2455 ± 15	0.7366 ± 0.0014
124150.1.1	ETH	Q4416	2463	2449 ± 15	0.7372 ± 0.0014
124151.1.1	ETH	Q4416	2462	2410 ± 15	0.7408 ± 0.0014
124152.1.1	ETH	Q4416	2461	2419 ± 15	0.7400 ± 0.0014
124153.1.1	ETH	Q4416	2460	2437 ± 15	0.7383 ± 0.0013
124154.1.1	ETH	Q4416	2459	2439 ± 15	0.7382 ± 0.0013
124155.1.1	ETH	Q4416	2458	2451 ± 15	0.7371 ± 0.0013
124156.1.1	ETH	Q4416	2457	2438 ± 15	0.7382 ± 0.0014
124157.1.1	ETH	Q4416	2456	2453 ± 15	0.7369 ± 0.0014
124158.1.1	ETH	Q4416	2455	2459 ± 15	0.7363 ± 0.0014
124159.1.1	ETH	Q4416	2454	2441 ± 15	0.7379 ± 0.0014
124160.1.1	ETH	Q4416	2453	2445 ± 15	0.7376 ± 0.0014
124161.1.1	ETH	Q4416	2452	2439 ± 15	0.7381 ± 0.0014
124162.1.1	ETH	Q4416	2451	2430 ± 15	0.7390 ± 0.0014
124163.1.1	ETH	Q4416	2450	2456 ± 15	0.7366 ± 0.0013
124163.1.2	ETH	Q4416	2450	2457 ± 12	0.7365 ± 0.0011
124164.1.2	ETH	Q4416	2449	2430 ± 12	0.7389 ± 0.0011
124165.1.2	ETH	Q4416	2448	2441 ± 12	0.7379 ± 0.0011
124166.1.2	ETH	Q4416	2447	2433 ± 12	0.7387 ± 0.0011
124167.1.2	ETH	Q4416	2446	2447 ± 12	0.7374 ± 0.0011
124168.1.2	ETH	Q4416	2445	2437 ± 12	0.7383 ± 0.0011
124169.1.2	ETH	Q4416	2444	2457 ± 12	0.7365 ± 0.0011
124170.1.2	ETH	Q4416	2443	2443 ± 12	0.7378 ± 0.0011
124171.1.1	ETH	Q4416	2442	2445 ± 14	0.7376 ± 0.0013
124171.1.2	ETH	Q4416	2442	2447 ± 12	0.7374 ± 0.0011
124172.1.1	ETH	Q4416	2441	2463 ± 14	0.7360 ± 0.0013

124172.1.2	ETH	Q4416	2441	2437 ± 12	0.7383 ± 0.0011
124173.1.1	ETH	Q4416	2440	2445 ± 14	0.7376 ± 0.0013
124174.1.1	ETH	Q4416	2439	2454 ± 14	0.7368 ± 0.0013
124175.1.1	ETH	Q4416	2438	2444 ± 14	0.7377 ± 0.0013
124176.1.1	ETH	Q4416	2437	2433 ± 14	0.7387 ± 0.0013
124177.1.1	ETH	Q4416	2436	2431 ± 14	0.7389 ± 0.0013
124178.1.1	ETH	Q4416	2435	2433 ± 14	0.7387 ± 0.0013
124179.1.1	ETH	Q4416	2434	2448 ± 14	0.7373 ± 0.0013
124180.1.1	ETH	Q4416	2433	2435 ± 14	0.7385 ± 0.0013
124181.1.1	ETH	Q4416	2432	2439 ± 14	0.7382 ± 0.0013
124182.1.1	ETH	Q4416	2431	2438 ± 14	0.7382 ± 0.0013
124183.1.1	ETH	Q4416	2430	2432 ± 14	0.7388 ± 0.0013
124184.1.1	ETH	Q4416	2429	2433 ± 14	0.7387 ± 0.0013
124185.1.1	ETH	Q4416	2428	2433 ± 14	0.7387 ± 0.0013
124186.1.1	ETH	Q4416	2427	2441 ± 14	0.7380 ± 0.0013
124187.1.1	ETH	Q4416	2426	2433 ± 14	0.7387 ± 0.0013
124188.1.1	ETH	Q4416	2425	2455 ± 14	0.7367 ± 0.0013
124189.1.1	ETH	Q4416	2424	2460 ± 14	0.7362 ± 0.0013
124190.1.1	ETH	Q4416	2423	2435 ± 14	0.7385 ± 0.0013
124191.1.1	ETH	Q4416	2422	2399 ± 14	0.7418 ± 0.0013
124192.1.1	ETH	Q4416	2421	2410 ± 14	0.7408 ± 0.0013
124192.1.2	ETH	Q4416	2421	2430 ± 15	0.7389 ± 0.0014
124193.1.1	ETH	Q4416	2420	2441 ± 14	0.7380 ± 0.0013
124194.1.1	ETH	Q4416	2419	2429 ± 14	0.7391 ± 0.0013
124194.1.2	ETH	Q4416	2419	2424 ± 15	0.7396 ± 0.0014
124195.1.1	ETH	Q4416	2418	2435 ± 14	0.7385 ± 0.0013
124196.1.2	ETH	Q4416	2417	2437 ± 16	0.7383 ± 0.0014
124197.1.2	ETH	Q4416	2416	2435 ± 16	0.7385 ± 0.0014
124198.1.2	ETH	Q4416	2415	2477 ± 16	0.7347 ± 0.0014
124199.1.2	ETH	Q4416	2414	2481 ± 16	0.7343 ± 0.0014
124200.1.2	ETH	Q4416	2413	2459 ± 16	0.7363 ± 0.0014
124201.1.2	ETH	Q4416	2412	2436 ± 16	0.7384 ± 0.0015
124203.1.1	ETH	Q4416	2410	2458 ± 14	0.7364 ± 0.0013
124204.1.1	ETH	Q4416	2409	2459 ± 14	0.7363 ± 0.0013
124204.1.2	ETH	Q4416	2409	2475 ± 16	0.7348 ± 0.0014
124205.1.1	ETH	Q4416	2408	2461 ± 14	0.7361 ± 0.0013
124206.1.1	ETH	Q4416	2407	2450 ± 14	0.7371 ± 0.0013
124207.1.1	ETH	Q4416	2406	2472 ± 14	0.7351 ± 0.0013
124207.1.2	ETH	Q4416	2406	2473 ± 16	0.7350 ± 0.0014
124208.1.1	ETH	Q4416	2405	2478 ± 14	0.7345 ± 0.0013
124209.1.1	ETH	Q4416	2404	2477 ± 14	0.7347 ± 0.0013
124209.1.2	ETH	Q4416	2404	2461 ± 16	0.7361 ± 0.0014
124210.1.1	ETH	Q4416	2403	2449 ± 14	0.7372 ± 0.0013
124210.1.2	ETH	Q4416	2403	2435 ± 16	0.7385 ± 0.0014
124211.1.1	ETH	Q4416	2402	2424 ± 14	0.7395 ± 0.0013
124211.1.2	ETH	Q4416	2402	2446 ± 16	0.7375 ± 0.0014
124212.1.1	ETH	Q4416	2401	2444 ± 14	0.7377 ± 0.0013
124213.1.1	ETH	Q4416	2400	2463 ± 14	0.7360 ± 0.0013
124213.1.2	ETH	Q4416	2400	2445 ± 16	0.7376 ± 0.0014
124214.1.1	ETH	Q4416	2399	2435 ± 14	0.7385 ± 0.0013
124214.1.2	ETH	Q4416	2399	2450 ± 16	0.7371 ± 0.0014
124215.1.1	ETH	Q4416	2398	2445 ± 14	0.7376 ± 0.0013
124215.1.2	ETH	Q4416	2398	2437 ± 16	0.7384 ± 0.0014
124216.1.1	ETH	Q4416	2397	2452 ± 14	0.7369 ± 0.0013
124217.1.1	ETH	Q4416	2396	2451 ± 14	0.7370 ± 0.0013
124217.1.2	ETH	Q4416	2396	2469 ± 16	0.7354 ± 0.0014
124218.1.1	ETH	Q4416	2395	2466 ± 14	0.7357 ± 0.0013
124219.1.1	ETH	Q4416	2394	2437 ± 14	0.7383 ± 0.0013
124219.1.2	ETH	Q4416	2394	2457 ± 16	0.7364 ± 0.0014

124220.1.1	ETH	Q4416	2393	2457 ± 14	0.7365 ± 0.0013
124220.1.2	ETH	Q4416	2393	2452 ± 16	0.7370 ± 0.0015
124221.1.1	ETH	Q4416	2392	2435 ± 14	0.7385 ± 0.0013
124222.1.1	ETH	Q4416	2391	2439 ± 14	0.7381 ± 0.0013
124223.1.1	ETH	Q4416	2390	2427 ± 14	0.7392 ± 0.0013
124224.1.1	ETH	Q4416	2389	2438 ± 15	0.7382 ± 0.0013
124225.1.1	ETH	Q4416	2388	2455 ± 14	0.7366 ± 0.0013
124226.1.1	ETH	Q4416	2387	2456 ± 15	0.7366 ± 0.0013
124226.1.2	ETH	Q4416	2387	2457 ± 16	0.7365 ± 0.0014
124227.1.1	ETH	Q4416	2386	2444 ± 15	0.7377 ± 0.0013
124228.1.1	ETH	Q4416	2385	2447 ± 14	0.7374 ± 0.0013
124228.1.2	ETH	Q4416	2385	2459 ± 15	0.7363 ± 0.0014
124229.1.1	ETH	Q4416	2384	2442 ± 15	0.7379 ± 0.0013
124229.1.2	ETH	Q4416	2384	2455 ± 16	0.7367 ± 0.0014
124230.1.1	ETH	Q4416	2383	2454 ± 15	0.7368 ± 0.0013
124231.1.1	ETH	Q4416	2382	2437 ± 15	0.7383 ± 0.0013
124232.1.1	ETH	Q4416	2381	2441 ± 15	0.7379 ± 0.0013
124232.1.2	ETH	Q4416	2381	2432 ± 16	0.7387 ± 0.0014
124233.1.1	ETH	Q4416	2380	2417 ± 14	0.7401 ± 0.0013
124234.1.1	ETH	Q4416	2379	2463 ± 14	0.7360 ± 0.0013
124234.1.2	ETH	Q4416	2379	2454 ± 16	0.7368 ± 0.0014
124235.1.1	ETH	Q4416	2378	2440 ± 14	0.7380 ± 0.0013
124235.1.2	ETH	Q4416	2378	2459 ± 16	0.7363 ± 0.0014
124236.1.1	ETH	Q4416	2377	2467 ± 14	0.7356 ± 0.0013
108880.1.1	ETH	gli-27	2350	2349 ± 16	0.7465 ± 0.0015
108880.1.2	ETH	gli-27	2350	2342 ± 15	0.7471 ± 0.0014
108881.1.1	ETH	gli-27	2349	2342 ± 16	0.7471 ± 0.0015
108882.1.1	ETH	gli-27	2348	2344 ± 16	0.7469 ± 0.0015
108883.1.1	ETH	gli-27	2347	2327 ± 16	0.7485 ± 0.0015
108883.1.2	ETH	gli-27	2347	2327 ± 15	0.7485 ± 0.0014
108884.1.1	ETH	gli-27	2346	2334 ± 16	0.7479 ± 0.0015
108885.1.1	ETH	gli-27	2345	2338 ± 16	0.7475 ± 0.0015
108886.1.1	ETH	gli-27	2344	2331 ± 16	0.7481 ± 0.0015
108886.1.2	ETH	gli-27	2344	2339 ± 15	0.7474 ± 0.0014
108887.1.1	ETH	gli-27	2343	2340 ± 16	0.7473 ± 0.0015
108888.1.1	ETH	gli-27	2342	2314 ± 16	0.7497 ± 0.0015
108889.1.1	ETH	gli-27	2341	2318 ± 16	0.7493 ± 0.0015
108889.1.2	ETH	gli-27	2341	2323 ± 15	0.7488 ± 0.0014
108890.1.1	ETH	gli-27	2340	2347 ± 16	0.7466 ± 0.0015
108891.1.1	ETH	gli-27	2339	2310 ± 16	0.7501 ± 0.0015
108892.1.1	ETH	gli-27	2338	2296 ± 16	0.7514 ± 0.0015
108892.1.2	ETH	gli-27	2338	2286 ± 15	0.7523 ± 0.0014
108893.1.1	ETH	gli-27	2337	2302 ± 16	0.7508 ± 0.0015
108894.1.1	ETH	gli-27	2336	2286 ± 16	0.7523 ± 0.0015
108895.1.1	ETH	gli-27	2335	2296 ± 16	0.7514 ± 0.0015
108895.1.2	ETH	gli-27	2335	2298 ± 15	0.7512 ± 0.0014
108896.1.1	ETH	gli-27	2334	2326 ± 16	0.7486 ± 0.0015
108897.1.1	ETH	gli-27	2333	2291 ± 16	0.7519 ± 0.0015
108898.1.1	ETH	gli-27	2332	2307 ± 17	0.7504 ± 0.0016
108898.1.2	ETH	gli-27	2332	2297 ± 15	0.7513 ± 0.0014
108899.1.1	ETH	gli-27	2331	2334 ± 17	0.7479 ± 0.0016
108900.1.1	ETH	gli-27	2330	2293 ± 16	0.7517 ± 0.0015
108901.1.1	ETH	gli-27	2329	2281 ± 16	0.7528 ± 0.0015
108901.1.2	ETH	gli-27	2329	2303 ± 15	0.7508 ± 0.0014
108902.1.1	ETH	gli-27	2328	2318 ± 17	0.7494 ± 0.0015
108903.1.1	ETH	gli-27	2327	2293 ± 16	0.7517 ± 0.0015
108904.1.1	ETH	gli-27	2326	2287 ± 16	0.7522 ± 0.0015
108904.1.2	ETH	gli-27	2326	2295 ± 15	0.7515 ± 0.0014
108905.1.1	ETH	gli-27	2325	2291 ± 13	0.7518 ± 0.0012

108906.1.1	ETH	gli-27	2324	2297 ± 13	0.7513 ± 0.0012
108907.1.1	ETH	gli-27	2323	2280 ± 13	0.7529 ± 0.0012
108907.1.2	ETH	gli-27	2323	2282 ± 15	0.7527 ± 0.0014
108908.1.1	ETH	gli-27	2322	2267 ± 13	0.7541 ± 0.0012
108909.1.1	ETH	gli-27	2321	2278 ± 13	0.7531 ± 0.0012
108910.1.1	ETH	gli-27	2320	2288 ± 13	0.7521 ± 0.0012
108910.1.2	ETH	gli-27	2320	2269 ± 15	0.7539 ± 0.0014
108911.1.1	ETH	gli-27	2319	2271 ± 13	0.7537 ± 0.0012
108912.1.1	ETH	gli-27	2318	2282 ± 13	0.7527 ± 0.0012
108913.1.1	ETH	gli-27	2317	2265 ± 13	0.7543 ± 0.0012
108913.1.2	ETH	gli-27	2317	2250 ± 15	0.7557 ± 0.0014
108914.1.1	ETH	gli-27	2316	2258 ± 13	0.7549 ± 0.0012
108915.1.1	ETH	gli-27	2315	2277 ± 13	0.7532 ± 0.0012
108916.1.1	ETH	gli-27	2314	2279 ± 13	0.7530 ± 0.0012
108916.1.2	ETH	gli-27	2314	2245 ± 15	0.7562 ± 0.0014
108917.1.1	ETH	gli-27	2313	2269 ± 13	0.7539 ± 0.0013
108918.1.1	ETH	gli-27	2312	2238 ± 13	0.7568 ± 0.0012
108919.1.1	ETH	gli-27	2311	2241 ± 13	0.7566 ± 0.0012
108919.1.2	ETH	gli-27	2311	2241 ± 15	0.7566 ± 0.0014
108920.1.1	ETH	gli-27	2310	2237 ± 13	0.7570 ± 0.0012
108921.1.1	ETH	gli-27	2309	2234 ± 13	0.7572 ± 0.0012
108922.1.1	ETH	gli-27	2308	2244 ± 13	0.7563 ± 0.0012
108922.1.2	ETH	gli-27	2308	2220 ± 15	0.7585 ± 0.0014
108923.1.1	ETH	gli-27	2307	2247 ± 14	0.7560 ± 0.0013
108924.1.1	ETH	gli-27	2306	2226 ± 13	0.7580 ± 0.0012
108925.1.1	ETH	gli-27	2305	2209 ± 13	0.7596 ± 0.0012
108925.1.2	ETH	gli-27	2305	2212 ± 15	0.7593 ± 0.0014
108926.1.1	ETH	gli-27	2304	2204 ± 13	0.7600 ± 0.0012
108927.1.1	ETH	gli-27	2303	2205 ± 13	0.7599 ± 0.0012
108928.1.1	ETH	gli-27	2302	2232 ± 13	0.7575 ± 0.0012
108928.1.2	ETH	gli-27	2302	2197 ± 15	0.7607 ± 0.0014
108929.1.1	ETH	gli-27	2301	2233 ± 13	0.7573 ± 0.0012
108930.1.1	ETH	gli-27	2300	2218 ± 15	0.7587 ± 0.0014
108931.1.1	ETH	gli-27	2299	2205 ± 15	0.7600 ± 0.0014
108931.1.3	ETH	gli-27	2299	2242 ± 15	0.7565 ± 0.0015
108932.1.1	ETH	gli-27	2298	2197 ± 15	0.7608 ± 0.0015
108933.1.1	ETH	gli-27	2297	2221 ± 15	0.7584 ± 0.0014
108934.1.1	ETH	gli-27	2296	2197 ± 15	0.7607 ± 0.0015
108934.1.3	ETH	gli-27	2296	2229 ± 15	0.7577 ± 0.0015
108935.1.1	ETH	gli-27	2295	2179 ± 15	0.7624 ± 0.0014
108936.1.1	ETH	gli-27	2294	2213 ± 15	0.7592 ± 0.0014
108937.1.1	ETH	gli-27	2293	2235 ± 15	0.7571 ± 0.0014
108937.1.3	ETH	gli-27	2293	2199 ± 15	0.7605 ± 0.0015
108938.1.1	ETH	gli-27	2292	2193 ± 15	0.7611 ± 0.0014
108939.1.1	ETH	gli-27	2291	2200 ± 15	0.7605 ± 0.0014
108940.1.1	ETH	gli-27	2290	2190 ± 15	0.7613 ± 0.0015
108940.1.3	ETH	gli-27	2290	2217 ± 16	0.7589 ± 0.0015
108941.1.1	ETH	gli-27	2289	2196 ± 16	0.7608 ± 0.0015
108942.1.1	ETH	gli-27	2288	2195 ± 16	0.7609 ± 0.0015
108943.1.1	ETH	gli-27	2287	2160 ± 16	0.7642 ± 0.0015
108943.1.3	ETH	gli-27	2287	2212 ± 15	0.7593 ± 0.0015
108944.1.1	ETH	gli-27	2286	2186 ± 15	0.7618 ± 0.0015
108945.1.2	ETH	gli-27	2285	2208 ± 15	0.7597 ± 0.0015
108946.1.1	ETH	ssm-48	2284	2185 ± 15	0.7618 ± 0.0014
108946.1.3	ETH	ssm-48	2284	2193 ± 15	0.7611 ± 0.0015
108947.1.1	ETH	ssm-48	2283	2178 ± 15	0.7625 ± 0.0014
108948.1.1	ETH	ssm-48	2282	2173 ± 15	0.7630 ± 0.0014
108949.1.1	ETH	ssm-48	2281	2193 ± 15	0.7611 ± 0.0014
108949.1.3	ETH	ssm-48	2281	2213 ± 15	0.7592 ± 0.0015

108950.1.1	ETH	ssm-48	2280	2160 ± 15	0.7642 ± 0.0015
108951.1.1	ETH	ssm-48	2279	2205 ± 15	0.7600 ± 0.0014
108952.1.1	ETH	ssm-48	2278	2170 ± 15	0.7633 ± 0.0014
108952.1.3	ETH	ssm-48	2278	2196 ± 15	0.7608 ± 0.0015
108953.1.1	ETH	ssm-48	2277	2159 ± 15	0.7643 ± 0.0014
108954.1.1	ETH	ssm-48	2276	2179 ± 15	0.7624 ± 0.0015
108955.1.1	ETH	ssm-48	2275	2194 ± 15	0.7610 ± 0.0014
108955.1.3	ETH	ssm-48	2275	2225 ± 16	0.7581 ± 0.0015
108956.1.1	ETH	ssm-48	2274	2174 ± 15	0.7629 ± 0.0014
108957.1.1	ETH	ssm-48	2273	2195 ± 15	0.7609 ± 0.0014
108958.1.1	ETH	ssm-48	2272	2190 ± 15	0.7614 ± 0.0014
108958.1.3	ETH	ssm-48	2272	2198 ± 16	0.7606 ± 0.0015
108959.1.1	ETH	ssm-48	2271	2220 ± 15	0.7585 ± 0.0014
108960.1.1	ETH	ssm-48	2270	2223 ± 15	0.7583 ± 0.0014
108961.1.1	ETH	ssm-48	2269	2209 ± 15	0.7596 ± 0.0014
108961.1.3	ETH	ssm-48	2269	2210 ± 15	0.7594 ± 0.0015
108962.1.1	ETH	ssm-48	2268	2202 ± 15	0.7602 ± 0.0014
108963.1.1	ETH	ssm-48	2267	2207 ± 15	0.7598 ± 0.0014
108964.1.1	ETH	ssm-48	2266	2263 ± 15	0.7545 ± 0.0014
108964.1.3	ETH	ssm-48	2266	2260 ± 15	0.7548 ± 0.0015
108965.1.1	ETH	ssm-48	2265	2219 ± 15	0.7586 ± 0.0014
108966.1.1	ETH	ssm-48	2264	2231 ± 15	0.7575 ± 0.0014
108967.1.1	ETH	ssm-48	2263	2233 ± 15	0.7573 ± 0.0014
108967.1.3	ETH	ssm-48	2263	2257 ± 15	0.7550 ± 0.0014
108968.1.1	ETH	ssm-48	2262	2221 ± 15	0.7585 ± 0.0014
108969.1.1	ETH	ssm-48	2261	2205 ± 15	0.7600 ± 0.0014
108970.1.1	ETH	ssm-48	2260	2200 ± 15	0.7604 ± 0.0014
108970.1.3	ETH	ssm-48	2260	2222 ± 15	0.7583 ± 0.0015
108971.1.1	ETH	ssm-48	2259	2206 ± 15	0.7599 ± 0.0014
108972.1.1	ETH	ssm-48	2258	2235 ± 15	0.7571 ± 0.0014
108973.1.1	ETH	ssm-48	2257	2216 ± 16	0.7589 ± 0.0015
108973.1.3	ETH	ssm-48	2257	2258 ± 15	0.7549 ± 0.0014
108974.1.1	ETH	ssm-48	2256	2236 ± 15	0.7570 ± 0.0014
108975.1.1	ETH	ssm-48	2255	2235 ± 15	0.7571 ± 0.0014
108976.1.1	ETH	ssm-48	2254	2232 ± 15	0.7574 ± 0.0014
108976.1.3	ETH	ssm-48	2254	2250 ± 15	0.7557 ± 0.0015
108977.1.1	ETH	ssm-48	2253	2231 ± 15	0.7575 ± 0.0014
108978.1.1	ETH	ssm-48	2252	2235 ± 15	0.7571 ± 0.0014
108979.1.1	ETH	ssm-48	2251	2243 ± 15	0.7563 ± 0.0014
108979.1.3	ETH	ssm-48	2251	2250 ± 15	0.7557 ± 0.0015
108980.1.1	ETH	ssm-48	2250	2263 ± 15	0.7545 ± 0.0014
108981.1.1	ETH	ssm-48	2249	2224 ± 16	0.7581 ± 0.0015
108982.1.1	ETH	ssm-48	2248	2235 ± 16	0.7571 ± 0.0015
108982.1.3	ETH	ssm-48	2248	2227 ± 15	0.7579 ± 0.0014
108983.1.1	ETH	ssm-48	2247	2247 ± 16	0.7560 ± 0.0015
108984.1.1	ETH	ssm-48	2246	2245 ± 16	0.7562 ± 0.0015
108985.1.1	ETH	ssm-48	2245	2235 ± 16	0.7571 ± 0.0015
108985.1.3	ETH	ssm-48	2245	2230 ± 15	0.7576 ± 0.0015
108986.1.1	ETH	ssm-48	2244	2195 ± 16	0.7609 ± 0.0015
108986.1.2	ETH	ssm-48	2244	2229 ± 14	0.7577 ± 0.0013
108987.1.1	ETH	ssm-48	2243	2237 ± 16	0.7569 ± 0.0015
108988.1.1	ETH	ssm-48	2242	2242 ± 16	0.7565 ± 0.0015
108988.1.3	ETH	ssm-48	2242	2229 ± 15	0.7577 ± 0.0014
108989.1.1	ETH	ssm-48	2241	2219 ± 16	0.7587 ± 0.0015
108990.1.1	ETH	ssm-48	2240	2218 ± 16	0.7588 ± 0.0015
108991.1.1	ETH	ssm-48	2239	2217 ± 16	0.7588 ± 0.0015
108991.1.3	ETH	ssm-48	2239	2238 ± 15	0.7568 ± 0.0014
108992.1.1	ETH	ssm-48	2238	2245 ± 16	0.7562 ± 0.0015
108993.1.1	ETH	ssm-48	2237	2233 ± 16	0.7573 ± 0.0015

108994.1.1	ETH	ssm-48	2236	2228 ± 16	0.7578 ± 0.0015
108994.1.3	ETH	ssm-48	2236	2232 ± 15	0.7574 ± 0.0015
108995.1.1	ETH	ssm-48	2235	2226 ± 16	0.7580 ± 0.0015
108996.1.1	ETH	ssm-48	2234	2244 ± 16	0.7563 ± 0.0015
108997.1.1	ETH	ssm-48	2233	2266 ± 16	0.7542 ± 0.0015
108997.1.3	ETH	ssm-48	2233	2239 ± 15	0.7568 ± 0.0014
108998.1.1	ETH	ssm-48	2232	2238 ± 16	0.7568 ± 0.0015
108999.1.1	ETH	ssm-48	2231	2218 ± 16	0.7587 ± 0.0015
109000.1.1	ETH	ssm-48	2230	2201 ± 16	0.7603 ± 0.0015
109000.1.3	ETH	ssm-48	2230	2209 ± 15	0.7596 ± 0.0015
109001.1.1	ETH	ssm-48	2229	2221 ± 16	0.7585 ± 0.0015
109002.1.1	ETH	ssm-48	2228	2231 ± 16	0.7575 ± 0.0015
109003.1.1	ETH	ssm-48	2227	2275 ± 16	0.7534 ± 0.0015
109003.1.3	ETH	ssm-48	2227	2271 ± 15	0.7538 ± 0.0014
109004.1.1	ETH	ssm-48	2226	2242 ± 17	0.7564 ± 0.0016
109005.1.1	ETH	ssm-48	2225	2235 ± 15	0.7571 ± 0.0014
109006.1.1	ETH	ssm-48	2224	2241 ± 15	0.7566 ± 0.0014
109006.1.3	ETH	ssm-48	2224	2253 ± 15	0.7555 ± 0.0014
109007.1.1	ETH	ssm-48	2223	2266 ± 15	0.7543 ± 0.0014
109008.1.1	ETH	ssm-48	2222	2220 ± 15	0.7585 ± 0.0014
109009.1.1	ETH	ssm-48	2221	2234 ± 15	0.7573 ± 0.0014
109009.1.3	ETH	ssm-48	2221	2268 ± 15	0.7540 ± 0.0014
109010.1.1	ETH	ssm-48	2220	2228 ± 15	0.7578 ± 0.0014
109011.1.1	ETH	ssm-48	2219	2224 ± 14	0.7581 ± 0.0014
109012.1.1	ETH	ssm-48	2218	2233 ± 15	0.7573 ± 0.0014
109012.1.2	ETH	ssm-48	2218	2195 ± 18	0.7609 ± 0.0017
109012.1.3	ETH	ssm-48	2218	2231 ± 14	0.7575 ± 0.0013
109013.1.1	ETH	ssm-48	2217	2235 ± 15	0.7571 ± 0.0014
109014.1.1	ETH	ssm-48	2216	2248 ± 15	0.7559 ± 0.0014
109015.1.1	ETH	ssm-48	2215	2257 ± 15	0.7551 ± 0.0014
109015.1.2	ETH	ssm-48	2215	2244 ± 16	0.7563 ± 0.0015
109016.1.1	ETH	ssm-48	2214	2242 ± 15	0.7565 ± 0.0014
109017.1.1	ETH	ssm-48	2213	2239 ± 15	0.7568 ± 0.0014
109018.1.1	ETH	ssm-48	2212	2254 ± 15	0.7554 ± 0.0014
109018.1.2	ETH	ssm-48	2212	2235 ± 16	0.7571 ± 0.0015
109019.1.1	ETH	ssm-48	2211	2235 ± 15	0.7571 ± 0.0014
109020.1.1	ETH	ssm-48	2210	2237 ± 14	0.7569 ± 0.0014
109021.1.1	ETH	ssm-48	2209	2215 ± 15	0.7590 ± 0.0014
109021.1.2	ETH	ssm-48	2209	2242 ± 16	0.7565 ± 0.0015
109022.1.1	ETH	ssm-48	2208	2250 ± 15	0.7557 ± 0.0014
109023.1.1	ETH	ssm-48	2207	2238 ± 15	0.7568 ± 0.0014
109024.1.1	ETH	ssm-48	2206	2245 ± 15	0.7562 ± 0.0014
109024.1.2	ETH	ssm-48	2206	2235 ± 16	0.7571 ± 0.0015
109025.1.1	ETH	ssm-48	2205	2261 ± 15	0.7547 ± 0.0014
109026.1.1	ETH	ssm-48	2204	2256 ± 15	0.7551 ± 0.0014
109027.1.1	ETH	ssm-48	2203	2251 ± 15	0.7556 ± 0.0014
109027.1.2	ETH	ssm-48	2203	2251 ± 16	0.7557 ± 0.0015
109028.1.1	ETH	ssm-48	2202	2234 ± 15	0.7573 ± 0.0014
109029.1.1	ETH	ssm-48	2201	2248 ± 15	0.7559 ± 0.0014
109030.1.1	ETH	ssm-48	2200	2267 ± 15	0.7541 ± 0.0014
109030.1.2	ETH	ssm-48	2200	2247 ± 16	0.7560 ± 0.0015
109031.1.1	ETH	ssm-48	2199	2233 ± 17	0.7573 ± 0.0016
109032.1.1	ETH	ssm-48	2198	2204 ± 17	0.7600 ± 0.0016
109033.1.1	ETH	ssm-48	2197	2240 ± 17	0.7566 ± 0.0016
109033.1.2	ETH	ssm-48	2197	2228 ± 16	0.7578 ± 0.0015
109034.1.1	ETH	ssm-48	2196	2224 ± 17	0.7582 ± 0.0016
109035.1.1	ETH	ssm-48	2195	2231 ± 17	0.7575 ± 0.0016
109036.1.1	ETH	ssm-48	2194	2241 ± 17	0.7565 ± 0.0016
109036.1.2	ETH	ssm-48	2194	2245 ± 16	0.7562 ± 0.0015

109037.1.1	ETH	ssm-48	2193	2246 ± 17	0.7561 ± 0.0016
109038.1.1	ETH	ssm-48	2192	2247 ± 17	0.7560 ± 0.0016
109039.1.1	ETH	ssm-48	2191	2209 ± 17	0.7596 ± 0.0016
109039.1.2	ETH	ssm-48	2191	2218 ± 16	0.7587 ± 0.0015
109040.1.1	ETH	ssm-48	2190	2214 ± 17	0.7592 ± 0.0016
109041.1.1	ETH	ssm-48	2189	2202 ± 17	0.7603 ± 0.0016
109042.1.1	ETH	ssm-48	2188	2206 ± 17	0.7598 ± 0.0016
109042.1.2	ETH	ssm-48	2188	2202 ± 16	0.7602 ± 0.0015
109043.1.1	ETH	ssm-48	2187	2196 ± 17	0.7608 ± 0.0016
109044.1.1	ETH	ssm-48	2186	2217 ± 17	0.7588 ± 0.0016
109045.1.1	ETH	ssm-48	2185	2202 ± 17	0.7602 ± 0.0016
109045.1.2	ETH	ssm-48	2185	2216 ± 16	0.7589 ± 0.0015
109046.1.1	ETH	ssm-48	2184	2195 ± 17	0.7609 ± 0.0016
109047.1.1	ETH	ssm-48	2183	2226 ± 17	0.7579 ± 0.0016
109048.1.1	ETH	ssm-48	2182	2214 ± 17	0.7591 ± 0.0016
109048.1.2	ETH	ssm-48	2182	2246 ± 17	0.7561 ± 0.0016
109049.1.1	ETH	ssm-48	2181	2213 ± 17	0.7592 ± 0.0016
109050.1.1	ETH	ssm-48	2180	2250 ± 18	0.7557 ± 0.0017
109051.1.1	ETH	ssm-48	2179	2213 ± 17	0.7592 ± 0.0016
109051.1.2	ETH	ssm-48	2179	2218 ± 16	0.7587 ± 0.0015
109052.1.1	ETH	ssm-48	2178	2203 ± 17	0.7602 ± 0.0016
109053.1.1	ETH	ssm-48	2177	2196 ± 17	0.7608 ± 0.0016
109054.1.1	ETH	ssm-48	2176	2188 ± 17	0.7616 ± 0.0016
109054.1.2	ETH	ssm-48	2176	2187 ± 16	0.7616 ± 0.0015
109055.1.1	ETH	ssm-48	2175	2178 ± 17	0.7625 ± 0.0016
109056.1.1	ETH	ssm-48	2174	2196 ± 14	0.7608 ± 0.0013
109056.1.2	ETH	ssm-48	2174	2227 ± 15	0.7579 ± 0.0014
109057.1.1	ETH	ssm-48	2173	2224 ± 14	0.7582 ± 0.0013
109057.1.2	ETH	ssm-48	2173	2216 ± 16	0.7589 ± 0.0015
109057.1.3	ETH	ssm-48	2173	2232 ± 15	0.7574 ± 0.0014
109058.1.1	ETH	ssm-48	2172	2229 ± 14	0.7577 ± 0.0013
109058.1.2	ETH	ssm-48	2172	2205 ± 15	0.7599 ± 0.0014
109059.1.1	ETH	ssm-48	2171	2217 ± 14	0.7588 ± 0.0013
109059.1.2	ETH	ssm-48	2171	2257 ± 15	0.7550 ± 0.0014
109060.1.1	ETH	ssm-48	2170	2220 ± 14	0.7586 ± 0.0013
109060.1.2	ETH	ssm-48	2170	2207 ± 16	0.7598 ± 0.0015
109060.1.3	ETH	ssm-48	2170	2229 ± 15	0.7577 ± 0.0014
109061.1.1	ETH	ssm-48	2169	2212 ± 14	0.7593 ± 0.0013
109061.1.2	ETH	ssm-48	2169	2235 ± 15	0.7572 ± 0.0014
109062.1.1	ETH	ssm-48	2168	2235 ± 14	0.7571 ± 0.0013
109062.1.2	ETH	ssm-48	2168	2245 ± 15	0.7562 ± 0.0014
109063.1.1	ETH	ssm-48	2167	2231 ± 14	0.7575 ± 0.0013
109063.1.3	ETH	ssm-48	2167	2228 ± 15	0.7578 ± 0.0014
109064.1.1	ETH	ssm-48	2166	2231 ± 14	0.7575 ± 0.0013
109064.1.2	ETH	ssm-48	2166	2233 ± 15	0.7573 ± 0.0014
109065.1.1	ETH	ssm-48	2165	2224 ± 14	0.7581 ± 0.0013
109065.1.2	ETH	ssm-48	2165	2228 ± 15	0.7578 ± 0.0014
109066.1.1	ETH	ssm-48	2164	2206 ± 14	0.7599 ± 0.0013
109066.1.2	ETH	ssm-48	2164	2201 ± 16	0.7603 ± 0.0016
109066.1.3	ETH	ssm-48	2164	2218 ± 15	0.7587 ± 0.0014
109067.1.1	ETH	ssm-48	2163	2211 ± 14	0.7594 ± 0.0013
109067.1.2	ETH	ssm-48	2163	2215 ± 15	0.7590 ± 0.0014
109068.1.1	ETH	ssm-48	2162	2251 ± 16	0.7556 ± 0.0015
109068.1.2	ETH	ssm-48	2162	2216 ± 15	0.7589 ± 0.0014
109069.1.1	ETH	ssm-48	2161	2213 ± 14	0.7592 ± 0.0013
109069.1.2	ETH	ssm-48	2161	2216 ± 16	0.7589 ± 0.0015
109069.1.3	ETH	ssm-48	2161	2222 ± 15	0.7583 ± 0.0014
109070.1.1	ETH	ssm-48	2160	2237 ± 14	0.7570 ± 0.0013
109070.1.2	ETH	ssm-48	2160	2253 ± 15	0.7554 ± 0.0014

109071.1.1	ETH	ssm-48	2159	2229 ± 14	0.7577 ± 0.0013
109071.1.2	ETH	ssm-48	2159	2215 ± 15	0.7590 ± 0.0014
109072.1.1	ETH	ssm-48	2158	2250 ± 14	0.7557 ± 0.0013
109072.1.2	ETH	ssm-48	2158	2217 ± 16	0.7588 ± 0.0015
109072.1.3	ETH	ssm-48	2158	2236 ± 15	0.7570 ± 0.0014
109073.1.1	ETH	ssm-48	2157	2209 ± 14	0.7596 ± 0.0013
109074.1.1	ETH	ssm-48	2156	2243 ± 14	0.7564 ± 0.0013
109075.1.1	ETH	ssm-48	2155	2223 ± 14	0.7583 ± 0.0013
109076.1.1	ETH	ssm-48	2154	2231 ± 14	0.7575 ± 0.0013
109077.1.1	ETH	ssm-48	2153	2236 ± 14	0.7570 ± 0.0013
109078.1.1	ETH	ssm-48	2152	2239 ± 14	0.7567 ± 0.0013
109079.1.2	ETH	ssm-48	2151	2246 ± 15	0.7561 ± 0.0014
109080.1.2	ETH	ssm-48	2150	2221 ± 15	0.7584 ± 0.0014
109081.1.1	ETH	ssm-48	2149	2192 ± 12	0.7611 ± 0.0011
109081.1.3	ETH	ssm-48	2149	2227 ± 15	0.7579 ± 0.0014
109082.1.1	ETH	ssm-48	2148	2194 ± 13	0.7610 ± 0.0012
109082.1.2	ETH	ssm-48	2148	2202 ± 15	0.7603 ± 0.0014
109083.1.1	ETH	ssm-48	2147	2206 ± 11	0.7599 ± 0.0011
109083.1.2	ETH	ssm-48	2147	2219 ± 15	0.7587 ± 0.0014
109084.1.1	ETH	ssm-48	2146	2197 ± 12	0.7607 ± 0.0011
109084.1.3	ETH	ssm-48	2146	2215 ± 15	0.7590 ± 0.0014
109085.1.1	ETH	ssm-48	2145	2184 ± 12	0.7619 ± 0.0011
109085.1.2	ETH	ssm-48	2145	2202 ± 15	0.7602 ± 0.0014
109086.1.1	ETH	ssm-48	2144	2169 ± 11	0.7634 ± 0.0011
109087.1.1	ETH	ssm-48	2143	2184 ± 12	0.7619 ± 0.0011
109087.1.2	ETH	ssm-48	2143	2215 ± 16	0.7590 ± 0.0015
109088.1.1	ETH	ssm-48	2142	2157 ± 11	0.7645 ± 0.0011
109089.1.1	ETH	ssm-48	2141	2209 ± 12	0.7596 ± 0.0011
109090.1.1	ETH	ssm-48	2140	2198 ± 12	0.7606 ± 0.0011
109090.1.2	ETH	ssm-48	2140	2202 ± 18	0.7602 ± 0.0017
109091.1.1	ETH	ssm-48	2139	2187 ± 12	0.7616 ± 0.0011
109092.1.1	ETH	ssm-48	2138	2174 ± 12	0.7629 ± 0.0011
109093.1.1	ETH	ssm-48	2137	2178 ± 11	0.7625 ± 0.0011
109093.1.2	ETH	ssm-48	2137	2204 ± 17	0.7601 ± 0.0016
109094.1.1	ETH	ssm-48	2136	2181 ± 12	0.7623 ± 0.0011
109095.1.1	ETH	ssm-48	2135	2178 ± 12	0.7625 ± 0.0011
109096.1.1	ETH	ssm-48	2134	2156 ± 12	0.7646 ± 0.0011
109096.1.2	ETH	ssm-48	2134	2161 ± 18	0.7641 ± 0.0017
109097.1.1	ETH	ssm-48	2133	2181 ± 12	0.7622 ± 0.0011
109098.1.1	ETH	ssm-48	2132	2154 ± 12	0.7648 ± 0.0011
109099.1.1	ETH	ssm-48	2131	2191 ± 12	0.7613 ± 0.0012
109100.1.1	ETH	ssm-48	2130	2202 ± 12	0.7603 ± 0.0011
109101.1.1	ETH	ssm-48	2129	2204 ± 13	0.7601 ± 0.0012
109102.1.1	ETH	ssm-48	2128	2171 ± 12	0.7632 ± 0.0011
111730.1.1	ETH	ssm-48	2127	2188 ± 16	0.7615 ± 0.0016
111730.1.2	ETH	ssm-48	2127	2176 ± 14	0.7627 ± 0.0014
111731.1.1	ETH	ssm-48	2126	2187 ± 16	0.7617 ± 0.0016
111732.1.1	ETH	ssm-48	2125	2159 ± 16	0.7643 ± 0.0016
111733.1.1	ETH	ssm-48	2124	2186 ± 16	0.7617 ± 0.0016
111733.1.2	ETH	ssm-48	2124	2176 ± 14	0.7628 ± 0.0013
111734.1.1	ETH	ssm-48	2123	2160 ± 16	0.7642 ± 0.0016
111735.1.1	ETH	ssm-48	2122	2146 ± 17	0.7656 ± 0.0016
111736.1.1	ETH	ssm-48	2121	2146 ± 16	0.7655 ± 0.0016
111736.1.2	ETH	ssm-48	2121	2146 ± 14	0.7656 ± 0.0014
111737.1.1	ETH	ssm-48	2120	2162 ± 16	0.7640 ± 0.0016
111738.1.1	ETH	ssm-48	2119	2149 ± 16	0.7653 ± 0.0016
111739.1.1	ETH	ssm-48	2118	2155 ± 16	0.7647 ± 0.0016
111739.1.2	ETH	ssm-48	2118	2145 ± 14	0.7656 ± 0.0014
111740.1.1	ETH	ssm-48	2117	2155 ± 16	0.7647 ± 0.0016

111741.1.1	ETH	vahu-1	2116	2149 ± 16	0.7653 ± 0.0016
111741.1.2	ETH	vahu-1	2116	2182 ± 15	0.7621 ± 0.0014
111742.1.1	ETH	vahu-1	2115	2148 ± 16	0.7653 ± 0.0016
111742.1.2	ETH	vahu-1	2115	2136 ± 14	0.7665 ± 0.0014
111742.1.3	ETH	vahu-1	2115	2186 ± 14	0.7617 ± 0.0014
111743.1.1	ETH	vahu-1	2114	2139 ± 16	0.7663 ± 0.0016
111743.1.2	ETH	vahu-1	2114	2170 ± 14	0.7633 ± 0.0014
111744.1.1	ETH	vahu-1	2113	2142 ± 16	0.7659 ± 0.0016
111744.1.2	ETH	vahu-1	2113	2172 ± 14	0.7631 ± 0.0014
111745.1.1	ETH	vahu-1	2112	2161 ± 16	0.7641 ± 0.0016
111745.1.2	ETH	vahu-1	2112	2141 ± 14	0.7660 ± 0.0014
111745.1.3	ETH	vahu-1	2112	2154 ± 14	0.7648 ± 0.0014
111746.1.1	ETH	vahu-1	2111	2131 ± 17	0.7670 ± 0.0016
111746.1.2	ETH	vahu-1	2111	2147 ± 15	0.7655 ± 0.0014
111747.1.1	ETH	vahu-1	2110	2153 ± 16	0.7649 ± 0.0016
111747.1.2	ETH	vahu-1	2110	2169 ± 15	0.7634 ± 0.0014
111748.1.1	ETH	vahu-1	2109	2134 ± 16	0.7667 ± 0.0016
111748.1.2	ETH	vahu-1	2109	2140 ± 14	0.7661 ± 0.0014
111748.1.3	ETH	vahu-1	2109	2144 ± 14	0.7657 ± 0.0014
111749.1.1	ETH	vahu-1	2108	2151 ± 16	0.7651 ± 0.0016
111749.1.2	ETH	vahu-1	2108	2155 ± 14	0.7647 ± 0.0014
111750.1.1	ETH	vahu-1	2107	2147 ± 16	0.7655 ± 0.0016
111750.1.2	ETH	vahu-1	2107	2128 ± 14	0.7672 ± 0.0014
111751.1.1	ETH	vahu-1	2106	2166 ± 17	0.7636 ± 0.0016
111751.1.2	ETH	vahu-1	2106	2136 ± 14	0.7665 ± 0.0014
111751.1.3	ETH	vahu-1	2106	2125 ± 14	0.7676 ± 0.0014
111751.1.4	ETH	vahu-1	2106	2121 ± 14	0.7680 ± 0.0014
111752.1.1	ETH	vahu-1	2105	2099 ± 14	0.7701 ± 0.0013
111752.1.2	ETH	vahu-1	2105	2115 ± 14	0.7685 ± 0.0013
111753.1.1	ETH	vahu-1	2104	2088 ± 14	0.7711 ± 0.0013
111753.1.2	ETH	vahu-1	2104	2119 ± 14	0.7681 ± 0.0013
111754.1.1	ETH	vahu-1	2103	2123 ± 14	0.7678 ± 0.0013
111754.1.2	ETH	vahu-1	2103	2129 ± 14	0.7672 ± 0.0014
111754.1.3	ETH	vahu-1	2103	2132 ± 14	0.7669 ± 0.0014
111755.1.1	ETH	vahu-1	2102	2111 ± 14	0.7689 ± 0.0013
111756.1.1	ETH	vahu-1	2101	2117 ± 14	0.7683 ± 0.0013
111756.1.2	ETH	vahu-1	2101	2092 ± 14	0.7708 ± 0.0014
111757.1.1	ETH	vahu-1	2100	2108 ± 14	0.7692 ± 0.0013
111757.1.2	ETH	vahu-1	2100	2131 ± 14	0.7670 ± 0.0014
111757.1.3	ETH	vahu-1	2100	2157 ± 14	0.7646 ± 0.0014
111758.1.1	ETH	vahu-1	2099	2115 ± 14	0.7685 ± 0.0013
111758.1.2	ETH	vahu-1	2099	2128 ± 14	0.7673 ± 0.0014
111759.1.1	ETH	vahu-1	2098	2114 ± 14	0.7686 ± 0.0013
111759.1.2	ETH	vahu-1	2098	2152 ± 14	0.7650 ± 0.0014
111760.1.1	ETH	vahu-1	2097	2127 ± 14	0.7674 ± 0.0013
111760.1.2	ETH	vahu-1	2097	2134 ± 14	0.7667 ± 0.0014
111760.1.3	ETH	vahu-1	2097	2144 ± 14	0.7657 ± 0.0014
111761.1.1	ETH	vahu-1	2096	2124 ± 14	0.7677 ± 0.0013
111761.1.2	ETH	vahu-1	2096	2147 ± 14	0.7655 ± 0.0014
111762.1.1	ETH	vahu-1	2095	2128 ± 14	0.7672 ± 0.0013
111762.1.2	ETH	vahu-1	2095	2140 ± 14	0.7661 ± 0.0014
111763.1.1	ETH	vahu-1	2094	2107 ± 14	0.7693 ± 0.0013
111763.1.2	ETH	vahu-1	2094	2138 ± 14	0.7663 ± 0.0014
111763.1.3	ETH	vahu-1	2094	2121 ± 14	0.7679 ± 0.0014
111764.1.1	ETH	vahu-1	2093	2098 ± 14	0.7701 ± 0.0013
111764.1.2	ETH	vahu-1	2093	2149 ± 14	0.7653 ± 0.0014
111765.1.1	ETH	vahu-1	2092	2108 ± 14	0.7692 ± 0.0013
111765.1.2	ETH	vahu-1	2092	2102 ± 14	0.7697 ± 0.0014
111766.1.1	ETH	vahu-1	2091	2101 ± 14	0.7699 ± 0.0013

111766.1.2	ETH	vahu-1	2091	2103 ± 14	0.7697 ± 0.0014
111766.1.3	ETH	vahu-1	2091	2096 ± 14	0.7704 ± 0.0014
111767.1.1	ETH	vahu-1	2090	2101 ± 14	0.7699 ± 0.0013
111767.1.2	ETH	vahu-1	2090	2104 ± 14	0.7696 ± 0.0014
111768.1.1	ETH	vahu-1	2089	2097 ± 14	0.7703 ± 0.0013
111769.1.1	ETH	vahu-1	2088	2096 ± 14	0.7703 ± 0.0013
111769.1.2	ETH	vahu-1	2088	2098 ± 14	0.7702 ± 0.0014
111770.1.1	ETH	vahu-1	2087	2101 ± 14	0.7699 ± 0.0014
111771.1.1	ETH	vahu-1	2086	2087 ± 14	0.7712 ± 0.0014
111772.1.1	ETH	vahu-1	2085	2078 ± 14	0.7720 ± 0.0013
111772.1.2	ETH	vahu-1	2085	2082 ± 14	0.7717 ± 0.0014
111773.1.1	ETH	vahu-1	2084	2066 ± 14	0.7732 ± 0.0013
111774.1.1	ETH	vahu-1	2083	2096 ± 14	0.7704 ± 0.0013
111775.1.1	ETH	vahu-1	2082	2087 ± 14	0.7712 ± 0.0013
111775.1.2	ETH	vahu-1	2082	2078 ± 14	0.7721 ± 0.0014
111776.1.1	ETH	vahu-1	2081	2100 ± 14	0.7699 ± 0.0013
111777.1.1	ETH	vahu-1	2080	2080 ± 14	0.7718 ± 0.0013
111778.1.1	ETH	vahu-1	2079	2096 ± 15	0.7703 ± 0.0015
111778.1.2	ETH	vahu-1	2079	2097 ± 14	0.7702 ± 0.0014
111779.1.1	ETH	vahu-1	2078	2120 ± 15	0.7681 ± 0.0015
111780.1.1	ETH	vahu-1	2077	2120 ± 15	0.7681 ± 0.0015
111781.1.1	ETH	vahu-1	2076	2120 ± 15	0.7680 ± 0.0014
111781.1.2	ETH	vahu-1	2076	2074 ± 14	0.7724 ± 0.0014
111782.1.1	ETH	vahu-1	2075	2116 ± 15	0.7684 ± 0.0014
111783.1.1	ETH	vahu-1	2074	2098 ± 15	0.7702 ± 0.0014
111784.1.1	ETH	vahu-1	2073	2117 ± 15	0.7683 ± 0.0014
111784.1.2	ETH	vahu-1	2073	2082 ± 14	0.7716 ± 0.0014
111785.1.1	ETH	vahu-1	2072	2112 ± 15	0.7688 ± 0.0014
111786.1.1	ETH	vahu-1	2071	2112 ± 15	0.7688 ± 0.0014
111787.1.1	ETH	vahu-1	2070	2113 ± 15	0.7688 ± 0.0014
111787.1.2	ETH	vahu-1	2070	2126 ± 14	0.7675 ± 0.0014
111788.1.1	ETH	vahu-1	2069	2108 ± 15	0.7692 ± 0.0015
111789.1.1	ETH	vahu-1	2068	2135 ± 15	0.7666 ± 0.0014
111790.1.1	ETH	vahu-1	2067	2108 ± 15	0.7692 ± 0.0015
111790.1.2	ETH	vahu-1	2067	2117 ± 14	0.7683 ± 0.0014
111791.1.1	ETH	vahu-1	2066	2096 ± 15	0.7703 ± 0.0015
111792.1.1	ETH	vahu-1	2065	2105 ± 15	0.7695 ± 0.0015
111793.1.1	ETH	vahu-1	2064	2107 ± 15	0.7693 ± 0.0014
111793.1.2	ETH	vahu-1	2064	2089 ± 14	0.7710 ± 0.0014
111794.1.1	ETH	vahu-1	2063	2103 ± 15	0.7697 ± 0.0014
111795.1.1	ETH	vahu-1	2062	2093 ± 15	0.7707 ± 0.0015
111796.1.1	ETH	vahu-1	2061	2114 ± 15	0.7686 ± 0.0015
111796.1.2	ETH	vahu-1	2061	2101 ± 14	0.7698 ± 0.0014
111797.1.1	ETH	vahu-1	2060	2113 ± 15	0.7687 ± 0.0015
111798.1.1	ETH	vahu-1	2059	2112 ± 15	0.7688 ± 0.0015
111799.1.1	ETH	vahu-1	2058	2131 ± 15	0.7670 ± 0.0014
111799.1.2	ETH	vahu-1	2058	2116 ± 14	0.7684 ± 0.0014
111800.1.1	ETH	vahu-1	2057	2130 ± 15	0.7671 ± 0.0014
111801.1.1	ETH	vahu-1	2056	2120 ± 15	0.7681 ± 0.0015
111802.1.1	ETH	vahu-1	2055	2114 ± 15	0.7686 ± 0.0014
111802.1.2	ETH	vahu-1	2055	2107 ± 14	0.7693 ± 0.0014
111803.1.1	ETH	vahu-1	2054	2124 ± 15	0.7676 ± 0.0014
111804.1.1	ETH	vahu-1	2053	2118 ± 15	0.7682 ± 0.0014
111805.1.1	ETH	vahu-1	2052	2101 ± 15	0.7699 ± 0.0014
111805.1.2	ETH	vahu-1	2052	2106 ± 14	0.7694 ± 0.0014
111806.1.1	ETH	vahu-1	2051	2124 ± 15	0.7677 ± 0.0014
111807.1.1	ETH	vahu-1	2050	2115 ± 15	0.7686 ± 0.0014
111808.1.1	ETH	vahu-1	2049	2104 ± 15	0.7696 ± 0.0014
111808.1.2	ETH	vahu-1	2049	2116 ± 14	0.7684 ± 0.0013

111809.1.1	ETH	vahu-1	2048	2142 ± 15	0.7660 ± 0.0014
111810.1.1	ETH	vahu-1	2047	2125 ± 16	0.7676 ± 0.0016
111811.1.1	ETH	vahu-1	2046	2132 ± 15	0.7669 ± 0.0014
111811.1.2	ETH	vahu-1	2046	2100 ± 14	0.7699 ± 0.0013
111812.1.1	ETH	vahu-1	2045	2138 ± 15	0.7663 ± 0.0014
111813.1.1	ETH	vahu-1	2044	2113 ± 15	0.7688 ± 0.0014
111814.1.1	ETH	vahu-1	2043	2100 ± 15	0.7699 ± 0.0014
111814.1.2	ETH	vahu-1	2043	2100 ± 14	0.7699 ± 0.0013
111815.1.1	ETH	vahu-1	2042	2104 ± 15	0.7696 ± 0.0014
111816.1.1	ETH	vahu-1	2041	2078 ± 15	0.7721 ± 0.0014
111817.1.1	ETH	vahu-1	2040	2074 ± 15	0.7724 ± 0.0014
111817.1.2	ETH	vahu-1	2040	2094 ± 14	0.7705 ± 0.0013
111818.1.1	ETH	vahu-1	2039	2091 ± 15	0.7708 ± 0.0014
111819.1.1	ETH	vahu-1	2038	2096 ± 15	0.7703 ± 0.0014
111820.1.1	ETH	vahu-1	2037	2099 ± 15	0.7701 ± 0.0014
111820.1.2	ETH	vahu-1	2037	2095 ± 14	0.7704 ± 0.0013
111821.1.1	ETH	vahu-1	2036	2118 ± 15	0.7682 ± 0.0014
111822.1.1	ETH	vahu-1	2035	2118 ± 15	0.7683 ± 0.0014
111823.1.1	ETH	vahu-1	2034	2111 ± 15	0.7689 ± 0.0014
111823.1.2	ETH	vahu-1	2034	2081 ± 14	0.7718 ± 0.0013
111824.1.1	ETH	vahu-1	2033	2091 ± 15	0.7708 ± 0.0014
111825.1.1	ETH	vahu-1	2032	2109 ± 15	0.7690 ± 0.0014
111826.1.1	ETH	vahu-1	2031	2104 ± 15	0.7696 ± 0.0014
111826.1.2	ETH	vahu-1	2031	2100 ± 14	0.7699 ± 0.0013
111827.1.1	ETH	vahu-1	2030	2114 ± 15	0.7686 ± 0.0014
111828.1.1	ETH	vahu-1	2029	2087 ± 15	0.7712 ± 0.0014
111829.1.1	ETH	vahu-1	2028	2135 ± 14	0.7666 ± 0.0013
111829.1.2	ETH	vahu-1	2028	2096 ± 13	0.7703 ± 0.0013
111830.1.1	ETH	vahu-1	2027	2099 ± 14	0.7700 ± 0.0013
111831.1.1	ETH	vahu-1	2026	2099 ± 14	0.7700 ± 0.0013
111832.1.1	ETH	vahu-1	2025	2094 ± 14	0.7705 ± 0.0014
111832.1.2	ETH	vahu-1	2025	2083 ± 14	0.7716 ± 0.0013
111833.1.1	ETH	vahu-1	2024	2102 ± 14	0.7698 ± 0.0013
111834.1.1	ETH	vahu-1	2023	2112 ± 14	0.7688 ± 0.0014
111835.1.1	ETH	vahu-1	2022	2103 ± 14	0.7697 ± 0.0013
111835.1.2	ETH	vahu-1	2022	2088 ± 14	0.7711 ± 0.0013
111836.1.1	ETH	vahu-1	2021	2067 ± 14	0.7731 ± 0.0013
111837.1.1	ETH	vahu-1	2020	2100 ± 14	0.7700 ± 0.0014
111838.1.1	ETH	vahu-1	2019	2085 ± 14	0.7714 ± 0.0013
111838.1.2	ETH	vahu-1	2019	2080 ± 14	0.7719 ± 0.0013
111839.1.1	ETH	vahu-1	2018	2101 ± 14	0.7699 ± 0.0013
111840.1.1	ETH	vahu-1	2017	2116 ± 14	0.7685 ± 0.0014
111841.1.1	ETH	vahu-1	2016	2091 ± 14	0.7708 ± 0.0014
111841.1.2	ETH	vahu-1	2016	2098 ± 14	0.7702 ± 0.0013
111842.1.1	ETH	vahu-1	2015	2095 ± 14	0.7704 ± 0.0013
111843.1.1	ETH	vahu-1	2014	2111 ± 14	0.7689 ± 0.0013
111844.1.1	ETH	vahu-1	2013	2085 ± 14	0.7714 ± 0.0014
111844.1.2	ETH	vahu-1	2013	2099 ± 14	0.7701 ± 0.0013
111845.1.1	ETH	vahu-1	2012	2091 ± 14	0.7708 ± 0.0013
111846.1.1	ETH	vahu-1	2011	2093 ± 14	0.7707 ± 0.0013
111847.1.1	ETH	vahu-1	2010	2080 ± 14	0.7719 ± 0.0013
111847.1.2	ETH	vahu-1	2010	2092 ± 14	0.7707 ± 0.0013
111848.1.1	ETH	tah-29	2009	2082 ± 14	0.7717 ± 0.0013
111849.1.1	ETH	tah-29	2008	2084 ± 14	0.7715 ± 0.0013
111850.1.1	ETH	tah-29	2007	2084 ± 14	0.7715 ± 0.0013
111850.1.2	ETH	tah-29	2007	2072 ± 14	0.7726 ± 0.0013
111851.1.1	ETH	tah-29	2006	2076 ± 14	0.7722 ± 0.0013
111852.1.1	ETH	tah-29	2005	2111 ± 14	0.7689 ± 0.0013
111853.1.1	ETH	tah-29	2004	2084 ± 14	0.7715 ± 0.0013

111853.1.2	ETH	tah-29	2004	2084 ± 13	0.7715 ± 0.0013
111854.1.1	ETH	tah-29	2003	2068 ± 15	0.7730 ± 0.0014
111855.1.1	ETH	tah-29	2002	2077 ± 15	0.7722 ± 0.0014
111856.1.1	ETH	tah-29	2001	2072 ± 15	0.7726 ± 0.0014
111856.1.2	ETH	tah-29	2001	2089 ± 14	0.7710 ± 0.0013
111857.1.1	ETH	tah-29	2000	2074 ± 15	0.7724 ± 0.0014
111858.1.1	ETH	tah-29	1999	2053 ± 15	0.7744 ± 0.0014
111859.1.1	ETH	tah-29	1998	2024 ± 15	0.7773 ± 0.0014
111859.1.2	ETH	tah-29	1998	2046 ± 14	0.7752 ± 0.0013
111860.1.1	ETH	tah-29	1997	2029 ± 15	0.7767 ± 0.0014
111861.1.1	ETH	tah-29	1996	2042 ± 15	0.7756 ± 0.0014
111862.1.1	ETH	tah-29	1995	2023 ± 15	0.7774 ± 0.0014
111862.1.2	ETH	tah-29	1995	2037 ± 13	0.7761 ± 0.0013
111863.1.1	ETH	tah-29	1994	2025 ± 15	0.7772 ± 0.0014
111864.1.1	ETH	tah-29	1993	2032 ± 15	0.7765 ± 0.0014
111865.1.1	ETH	tah-29	1992	2007 ± 15	0.7790 ± 0.0014
111865.1.2	ETH	tah-29	1992	2049 ± 14	0.7748 ± 0.0013
111866.1.1	ETH	tah-29	1991	2030 ± 15	0.7767 ± 0.0015
111867.1.1	ETH	tah-29	1990	2032 ± 15	0.7765 ± 0.0014
111868.1.1	ETH	tah-29	1989	2040 ± 15	0.7757 ± 0.0014
111868.1.2	ETH	tah-29	1989	2018 ± 13	0.7779 ± 0.0013
111869.1.1	ETH	tah-29	1988	2017 ± 15	0.7780 ± 0.0014
111870.1.1	ETH	tah-29	1987	2002 ± 15	0.7794 ± 0.0014
111871.1.1	ETH	tah-29	1986	2001 ± 15	0.7795 ± 0.0014
111871.1.2	ETH	tah-29	1986	2016 ± 14	0.7781 ± 0.0013
111872.1.1	ETH	tah-29	1985	2013 ± 15	0.7784 ± 0.0014
111873.1.1	ETH	tah-29	1984	1984 ± 15	0.7811 ± 0.0014
111874.1.1	ETH	tah-29	1983	1986 ± 15	0.7810 ± 0.0014
111874.1.2	ETH	tah-29	1983	1997 ± 14	0.7799 ± 0.0013
111875.1.1	ETH	tah-29	1982	1976 ± 15	0.7819 ± 0.0015
111876.1.1	ETH	tah-29	1981	1994 ± 15	0.7802 ± 0.0014
111877.1.1	ETH	tah-29	1980	1988 ± 19	0.7807 ± 0.0018
111877.1.2	ETH	tah-29	1980	2022 ± 13	0.7775 ± 0.0013
111878.1.1	ETH	tah-29	1979	2003 ± 19	0.7793 ± 0.0019
111879.1.1	ETH	tah-29	1978	1988 ± 19	0.7808 ± 0.0019
111880.1.1	ETH	tah-29	1977	1968 ± 18	0.7827 ± 0.0018
111880.1.2	ETH	tah-29	1977	2002 ± 13	0.7794 ± 0.0013
111881.1.1	ETH	tah-29	1976	2015 ± 19	0.7782 ± 0.0019
111882.1.1	ETH	tah-29	1975	1989 ± 19	0.7807 ± 0.0018
111883.1.1	ETH	tah-29	1974	1990 ± 19	0.7806 ± 0.0019
111883.1.2	ETH	tah-29	1974	1994 ± 13	0.7802 ± 0.0013
111884.1.1	ETH	tah-29	1973	2016 ± 19	0.7781 ± 0.0018
111885.1.1	ETH	tah-29	1972	2007 ± 16	0.7789 ± 0.0015
111886.1.1	ETH	tah-29	1971	2025 ± 16	0.7772 ± 0.0016
111886.1.2	ETH	tah-29	1971	2043 ± 16	0.7755 ± 0.0015
111887.1.1	ETH	tah-29	1970	2000 ± 16	0.7796 ± 0.0015
111888.1.1	ETH	tah-29	1969	2023 ± 16	0.7774 ± 0.0015
111889.1.1	ETH	tah-29	1968	2010 ± 16	0.7786 ± 0.0015
111889.1.2	ETH	tah-29	1968	2020 ± 16	0.7777 ± 0.0015
111890.1.1	ETH	tah-29	1967	2027 ± 16	0.7770 ± 0.0015
111891.1.1	ETH	tah-29	1966	2019 ± 16	0.7777 ± 0.0015
111892.1.1	ETH	tah-29	1965	2031 ± 16	0.7766 ± 0.0015
111892.1.2	ETH	tah-29	1965	2020 ± 16	0.7777 ± 0.0015
111893.1.1	ETH	tah-29	1964	2024 ± 16	0.7773 ± 0.0015
111894.1.1	ETH	tah-29	1963	2050 ± 16	0.7747 ± 0.0015
111895.1.1	ETH	tah-29	1962	2041 ± 16	0.7756 ± 0.0015
111895.1.2	ETH	tah-29	1962	2049 ± 16	0.7749 ± 0.0016
111896.1.1	ETH	tah-29	1961	2050 ± 16	0.7747 ± 0.0015
111897.1.1	ETH	tah-29	1960	2042 ± 16	0.7755 ± 0.0015

111898.1.1	ETH	tah-29	1959	2035 ± 16	0.7762 ± 0.0015
111898.1.2	ETH	tah-29	1959	2059 ± 15	0.7739 ± 0.0015
111899.1.1	ETH	tah-29	1958	2024 ± 16	0.7773 ± 0.0015
111900.1.1	ETH	tah-29	1957	2023 ± 16	0.7774 ± 0.0015
111901.1.1	ETH	tah-29	1956	2019 ± 16	0.7778 ± 0.0015
111901.1.2	ETH	tah-29	1956	2018 ± 15	0.7779 ± 0.0014
111902.1.1	ETH	tah-29	1955	2017 ± 16	0.7779 ± 0.0015
111903.1.1	ETH	tah-29	1954	2012 ± 16	0.7784 ± 0.0015
111904.1.1	ETH	tah-29	1953	2014 ± 16	0.7783 ± 0.0015
111904.1.2	ETH	tah-29	1953	2016 ± 15	0.7780 ± 0.0014
111905.1.1	ETH	tah-29	1952	2009 ± 16	0.7787 ± 0.0015
111906.1.1	ETH	tah-29	1951	2023 ± 16	0.7774 ± 0.0015
112212.1.1	ETH	tah-29	2012	2077 ± 15	0.7722 ± 0.0014
112213.1.1	ETH	tah-29	2011	2062 ± 15	0.7736 ± 0.0015
112214.1.1	ETH	tah-29	2010	2081 ± 15	0.7717 ± 0.0014
126809.1.1	ETH	gp-22	2800	2749 ± 13	0.7102 ± 0.0011
126809.1.2	ETH	gp-22	2800	2731 ± 14	0.7117 ± 0.0012
126810.1.1	ETH	gp-22	2801	2733 ± 12	0.7116 ± 0.0011
126811.1.1	ETH	gp-22	2802	2734 ± 12	0.7115 ± 0.0011
126812.1.1	ETH	gp-22	2803	2731 ± 12	0.7118 ± 0.0011
126812.1.2	ETH	gp-22	2803	2732 ± 14	0.7117 ± 0.0012
126813.1.1	ETH	gp-22	2804	2726 ± 12	0.7123 ± 0.0011
126814.1.1	ETH	gp-22	2805	2745 ± 12	0.7106 ± 0.0011
126815.1.1	ETH	gp-22	2806	2751 ± 13	0.7100 ± 0.0011
126815.1.2	ETH	gp-22	2806	2719 ± 14	0.7128 ± 0.0012
126816.1.1	ETH	gp-22	2807	2756 ± 12	0.7096 ± 0.0011
126817.1.1	ETH	gp-22	2808	2754 ± 12	0.7097 ± 0.0011
126818.1.1	ETH	gp-22	2809	2770 ± 13	0.7084 ± 0.0011
126818.1.2	ETH	gp-22	2809	2761 ± 14	0.7092 ± 0.0012
126819.1.1	ETH	gp-22	2810	2753 ± 13	0.7098 ± 0.0011
126820.1.1	ETH	gp-22	2811	2745 ± 12	0.7106 ± 0.0011
126821.1.1	ETH	gp-22	2812	2746 ± 12	0.7104 ± 0.0011
126821.1.2	ETH	gp-22	2812	2739 ± 14	0.7110 ± 0.0012
126822.1.1	ETH	gp-22	2813	2736 ± 13	0.7114 ± 0.0011
126823.1.1	ETH	gp-22	2814	2750 ± 13	0.7101 ± 0.0011
126824.1.1	ETH	gp-22	2815	2746 ± 12	0.7104 ± 0.0011
126824.1.2	ETH	gp-22	2815	2741 ± 14	0.7109 ± 0.0012
126825.1.1	ETH	gp-22	2816	2738 ± 12	0.7112 ± 0.0011
126826.1.1	ETH	gp-22	2817	2736 ± 13	0.7114 ± 0.0011
126827.1.1	ETH	gp-22	2818	2725 ± 12	0.7123 ± 0.0011
126827.1.2	ETH	gp-22	2818	2738 ± 14	0.7112 ± 0.0012
126828.1.1	ETH	gp-22	2819	2729 ± 12	0.7120 ± 0.0011
126829.1.1	ETH	gp-22	2820	2728 ± 12	0.7120 ± 0.0011
126830.1.1	ETH	gp-22	2821	2722 ± 12	0.7126 ± 0.0011
126830.1.2	ETH	gp-22	2821	2729 ± 14	0.7120 ± 0.0012
126830.1.3	ETH	gp-22	2821	2722 ± 14	0.7126 ± 0.0012
126831.1.1	ETH	gp-22	2822	2724 ± 12	0.7124 ± 0.0011
126832.1.1	ETH	gp-22	2823	2734 ± 13	0.7115 ± 0.0011
126833.1.1	ETH	gp-22	2824	2732 ± 12	0.7117 ± 0.0011
126833.1.2	ETH	gp-22	2824	2731 ± 14	0.7118 ± 0.0012
126834.1.1	ETH	gp-22	2825	2726 ± 13	0.7122 ± 0.0011
126835.1.1	ETH	gp-22	2826	2735 ± 13	0.7114 ± 0.0011
126836.1.1	ETH	gp-22	2827	2739 ± 13	0.7111 ± 0.0011
126836.1.2	ETH	gp-22	2827	2736 ± 14	0.7113 ± 0.0012
126837.1.1	ETH	gp-22	2828	2718 ± 13	0.7129 ± 0.0011
126838.1.1	ETH	gp-22	2829	2734 ± 13	0.7115 ± 0.0011
126839.1.1	ETH	gp-22	2830	2733 ± 13	0.7116 ± 0.0011
126839.1.2	ETH	gp-22	2830	2737 ± 14	0.7113 ± 0.0012
126840.1.1	ETH	gp-22	2831	2734 ± 13	0.7116 ± 0.0011

126841.1.1	ETH	gp-22	2832	2716 ± 13	0.7131 ± 0.0011
126842.1.1	ETH	gp-22	2833	2724 ± 13	0.7124 ± 0.0011
126842.1.2	ETH	gp-22	2833	2719 ± 14	0.7129 ± 0.0012
126843.1.1	ETH	gp-22	2834	2717 ± 13	0.7130 ± 0.0011
126844.1.1	ETH	gp-22	2835	2726 ± 13	0.7122 ± 0.0011
126845.1.1	ETH	gp-22	2836	2726 ± 13	0.7122 ± 0.0011
126845.1.2	ETH	gp-22	2836	2725 ± 14	0.7124 ± 0.0012
126846.1.1	ETH	gp-22	2837	2728 ± 13	0.7120 ± 0.0011
126847.1.1	ETH	gp-22	2838	2733 ± 13	0.7117 ± 0.0011
126848.1.1	ETH	gp-22	2839	2715 ± 13	0.7132 ± 0.0011
126848.1.2	ETH	gp-22	2839	2729 ± 14	0.7120 ± 0.0012
126849.1.1	ETH	gp-22	2840	2713 ± 13	0.7134 ± 0.0011
126850.1.1	ETH	gp-22	2841	2712 ± 13	0.7135 ± 0.0011
126851.1.1	ETH	gp-22	2842	2720 ± 13	0.7128 ± 0.0011
126851.1.2	ETH	gp-22	2842	2720 ± 14	0.7128 ± 0.0012
126852.1.1	ETH	gp-22	2843	2729 ± 13	0.7120 ± 0.0011
126853.1.1	ETH	gp-22	2844	2729 ± 13	0.7120 ± 0.0011
126854.1.1	ETH	gp-22	2845	2722 ± 13	0.7126 ± 0.0011
126854.1.2	ETH	gp-22	2845	2720 ± 14	0.7128 ± 0.0012
126855.1.1	ETH	gp-22	2846	2747 ± 13	0.7104 ± 0.0011
126856.1.1	ETH	gp-22	2847	2752 ± 13	0.7099 ± 0.0011
126857.1.1	ETH	gp-22	2848	2742 ± 13	0.7108 ± 0.0011
126857.1.2	ETH	gp-22	2848	2735 ± 14	0.7114 ± 0.0012
126858.1.1	ETH	gp-22	2849	2753 ± 13	0.7099 ± 0.0011
126859.1.1	ETH	gp-22	2850	2758 ± 13	0.7094 ± 0.0011
126860.1.1	ETH	gp-22	2851	2757 ± 13	0.7095 ± 0.0011
126860.1.2	ETH	gp-22	2851	2779 ± 14	0.7076 ± 0.0012
126861.1.1	ETH	gp-22	2852	2787 ± 16	0.7069 ± 0.0014
126862.1.1	ETH	gp-22	2853	2786 ± 16	0.7070 ± 0.0014
126863.1.1	ETH	gp-22	2854	2797 ± 16	0.7060 ± 0.0014
126863.1.2	ETH	gp-22	2854	2766 ± 14	0.7087 ± 0.0012
126864.1.1	ETH	gp-22	2855	2803 ± 16	0.7054 ± 0.0014
126865.1.1	ETH	gp-22	2856	2805 ± 16	0.7053 ± 0.0014
126866.1.1	ETH	gp-22	2857	2793 ± 16	0.7063 ± 0.0014
126866.1.2	ETH	gp-22	2857	2780 ± 14	0.7075 ± 0.0012
126867.1.1	ETH	gp-22	2858	2787 ± 16	0.7068 ± 0.0014
126868.1.1	ETH	gp-22	2859	2792 ± 16	0.7064 ± 0.0014
126869.1.1	ETH	gp-22	2860	2797 ± 16	0.7059 ± 0.0014
126869.1.2	ETH	gp-22	2860	2762 ± 14	0.7091 ± 0.0012
126870.1.1	ETH	gp-22	2861	2797 ± 16	0.7060 ± 0.0014
126871.1.1	ETH	gp-22	2862	2797 ± 16	0.7059 ± 0.0014
126872.1.1	ETH	gp-22	2863	2808 ± 16	0.7050 ± 0.0014
126872.1.2	ETH	gp-22	2863	2831 ± 14	0.7030 ± 0.0012
126873.1.1	ETH	gp-22	2864	2822 ± 16	0.7037 ± 0.0014
126874.1.1	ETH	gp-22	2865	2835 ± 16	0.7026 ± 0.0014
126875.1.1	ETH	gp-22	2866	2824 ± 16	0.7036 ± 0.0014
126875.1.2	ETH	gp-22	2866	2802 ± 14	0.7055 ± 0.0012
126876.1.1	ETH	gp-22	2867	2855 ± 16	0.7009 ± 0.0014
126877.1.1	ETH	gp-22	2868	2840 ± 16	0.7022 ± 0.0014
126878.1.1	ETH	gp-22	2869	2874 ± 16	0.6992 ± 0.0014
126878.1.2	ETH	gp-22	2869	2817 ± 14	0.7042 ± 0.0012
126879.1.1	ETH	gp-22	2870	2839 ± 16	0.7023 ± 0.0014
126880.1.1	ETH	gp-22	2871	2841 ± 16	0.7021 ± 0.0014
126881.1.1	ETH	gp-22	2872	2841 ± 16	0.7021 ± 0.0014
126881.1.2	ETH	gp-22	2872	2838 ± 14	0.7023 ± 0.0012
126882.1.1	ETH	gp-22	2873	2833 ± 16	0.7028 ± 0.0014
126883.1.1	ETH	gp-22	2874	2821 ± 16	0.7039 ± 0.0014
126884.1.1	ETH	gp-22	2875	2810 ± 16	0.7049 ± 0.0014
126884.1.2	ETH	gp-22	2875	2814 ± 14	0.7045 ± 0.0012

126885.1.1	ETH	gp-22	2876	2826 ± 16	0.7034 ± 0.0014
126886.1.1	ETH	gp-22	2877	2856 ± 16	0.7008 ± 0.0014
126887.1.1	ETH	gp-22	2878	2838 ± 14	0.7024 ± 0.0012
126887.1.2	ETH	gp-22	2878	2829 ± 14	0.7032 ± 0.0012
126888.1.1	ETH	gp-22	2879	2819 ± 14	0.7041 ± 0.0012
126889.1.1	ETH	gp-22	2880	2827 ± 14	0.7033 ± 0.0012
126890.1.1	ETH	gp-22	2881	2812 ± 14	0.7047 ± 0.0012
126890.1.2	ETH	gp-22	2881	2812 ± 14	0.7047 ± 0.0012
126891.1.1	ETH	gp-22	2882	2798 ± 14	0.7059 ± 0.0012
126892.1.1	ETH	gp-22	2883	2799 ± 14	0.7058 ± 0.0012
126893.1.1	ETH	gp-22	2884	2793 ± 14	0.7063 ± 0.0012
126893.1.2	ETH	gp-22	2884	2804 ± 14	0.7053 ± 0.0012
126894.1.1	ETH	gp-22	2885	2804 ± 14	0.7053 ± 0.0012
126895.1.1	ETH	gp-22	2886	2803 ± 14	0.7055 ± 0.0012
126896.1.1	ETH	hib-26	2887	2823 ± 14	0.7037 ± 0.0012
126896.1.2	ETH	hib-26	2887	2845 ± 14	0.7017 ± 0.0012
126897.1.1	ETH	hib-26	2888	2819 ± 14	0.7040 ± 0.0013
126898.1.1	ETH	hib-26	2889	2834 ± 14	0.7027 ± 0.0012
126899.1.1	ETH	hib-26	2890	2853 ± 14	0.7011 ± 0.0012
126899.1.2	ETH	hib-26	2890	2845 ± 14	0.7017 ± 0.0012
126900.1.1	ETH	hib-26	2891	2860 ± 14	0.7004 ± 0.0013
126901.1.1	ETH	hib-26	2892	2850 ± 14	0.7013 ± 0.0013
126902.1.1	ETH	hib-26	2893	2834 ± 14	0.7028 ± 0.0013
126902.1.2	ETH	hib-26	2893	2889 ± 14	0.6979 ± 0.0012
126903.1.1	ETH	hib-26	2894	2860 ± 14	0.7005 ± 0.0012
126904.1.1	ETH	hib-26	2895	2841 ± 14	0.7021 ± 0.0013
126905.1.1	ETH	hib-26	2896	2836 ± 14	0.7025 ± 0.0013
126905.1.2	ETH	hib-26	2896	2862 ± 14	0.7003 ± 0.0012
126906.1.1	ETH	hib-26	2897	2848 ± 14	0.7015 ± 0.0013
126907.1.1	ETH	hib-26	2898	2833 ± 14	0.7028 ± 0.0013
126908.1.1	ETH	hib-26	2899	2836 ± 14	0.7026 ± 0.0013
126908.1.2	ETH	hib-26	2899	2870 ± 14	0.6996 ± 0.0012
126909.1.1	ETH	hib-26	2900	2832 ± 14	0.7029 ± 0.0013
126910.1.1	ETH	hib-26	2901	2832 ± 14	0.7029 ± 0.0012
126911.1.1	ETH	hib-26	2902	2819 ± 14	0.7040 ± 0.0013
126911.1.2	ETH	hib-26	2902	2856 ± 14	0.7008 ± 0.0012
126912.1.1	ETH	hib-26	2903	2805 ± 14	0.7052 ± 0.0013
126913.1.1	ETH	hib-26	2904	2803 ± 18	0.7054 ± 0.0016
126913.1.2	ETH	hib-26	2904	2835 ± 14	0.7026 ± 0.0012
126914.1.1	ETH	hib-26	2905	2818 ± 18	0.7041 ± 0.0016
126915.1.1	ETH	hib-26	2906	2826 ± 18	0.7034 ± 0.0016
126916.1.1	ETH	hib-26	2907	2839 ± 18	0.7023 ± 0.0016
126916.1.2	ETH	hib-26	2907	2839 ± 14	0.7023 ± 0.0012
126917.1.1	ETH	hib-26	2908	2807 ± 18	0.7051 ± 0.0016
126918.1.1	ETH	hib-26	2909	2819 ± 18	0.7041 ± 0.0016
126919.1.1	ETH	hib-26	2910	2815 ± 18	0.7044 ± 0.0016
126919.1.2	ETH	hib-26	2910	2848 ± 14	0.7015 ± 0.0012
126920.1.1	ETH	hib-26	2911	2821 ± 18	0.7039 ± 0.0016
126921.1.1	ETH	hib-26	2912	2821 ± 18	0.7039 ± 0.0016
126921.1.2	ETH	hib-26	2912	2841 ± 14	0.7021 ± 0.0012
126922.1.1	ETH	hib-26	2913	2786 ± 18	0.7069 ± 0.0016
126923.1.1	ETH	hib-26	2914	2830 ± 18	0.7030 ± 0.0016
126924.1.1	ETH	hib-26	2915	2811 ± 18	0.7047 ± 0.0016
126924.1.2	ETH	hib-26	2915	2837 ± 14	0.7024 ± 0.0012
126925.1.1	ETH	hib-26	2916	2786 ± 18	0.7070 ± 0.0016
126926.1.1	ETH	hib-26	2917	2808 ± 18	0.7050 ± 0.0016
126927.1.1	ETH	hib-26	2918	2822 ± 18	0.7038 ± 0.0016
126927.1.2	ETH	hib-26	2918	2826 ± 14	0.7034 ± 0.0012
126928.1.1	ETH	hib-26	2919	2793 ± 18	0.7064 ± 0.0016

126929.1.1	ETH	hib-26	2920	2811 ± 18	0.7048 ± 0.0016
126930.1.1	ETH	hib-26	2921	2797 ± 18	0.7060 ± 0.0016
126930.1.2	ETH	hib-26	2921	2823 ± 14	0.7037 ± 0.0012
126931.1.1	ETH	hib-26	2922	2805 ± 18	0.7053 ± 0.0016
126932.1.1	ETH	hib-26	2923	2806 ± 18	0.7052 ± 0.0016
126933.1.1	ETH	hib-26	2924	2806 ± 18	0.7051 ± 0.0016
126933.1.2	ETH	hib-26	2924	2832 ± 14	0.7029 ± 0.0012
126934.1.1	ETH	hib-26	2925	2815 ± 18	0.7044 ± 0.0016
126935.1.1	ETH	hib-26	2926	2795 ± 18	0.7061 ± 0.0016
126936.1.1	ETH	hib-26	2927	2795 ± 18	0.7062 ± 0.0016
126936.1.2	ETH	hib-26	2927	2825 ± 13	0.7035 ± 0.0012
126937.1.1	ETH	hib-26	2928	2815 ± 18	0.7044 ± 0.0016
126938.1.1	ETH	hib-26	2929	2809 ± 18	0.7049 ± 0.0016
126939.1.2	ETH	hib-26	2930	2813 ± 14	0.7046 ± 0.0012
126939.1.3	ETH	hib-26	2930	2788 ± 14	0.7068 ± 0.0012
126940.1.2	ETH	hib-26	2931	2816 ± 14	0.7043 ± 0.0012
126941.1.2	ETH	hib-26	2932	2814 ± 14	0.7045 ± 0.0012
126942.1.2	ETH	hib-26	2933	2818 ± 14	0.7041 ± 0.0012
126942.1.3	ETH	hib-26	2933	2816 ± 14	0.7043 ± 0.0012
126943.1.2	ETH	hib-26	2934	2810 ± 14	0.7048 ± 0.0012
126944.1.2	ETH	hib-26	2935	2795 ± 14	0.7061 ± 0.0012
126945.1.2	ETH	hib-26	2936	2812 ± 14	0.7047 ± 0.0012
126945.1.3	ETH	hib-26	2936	2805 ± 14	0.7053 ± 0.0012
126946.1.2	ETH	hib-26	2937	2807 ± 14	0.7051 ± 0.0012
126947.1.2	ETH	hib-26	2938	2809 ± 14	0.7049 ± 0.0012
126948.1.2	ETH	hib-26	2939	2826 ± 14	0.7034 ± 0.0012
126948.1.3	ETH	hib-26	2939	2820 ± 14	0.7040 ± 0.0012
126949.1.2	ETH	hib-26	2940	2837 ± 14	0.7024 ± 0.0012
126950.1.2	ETH	hib-26	2941	2834 ± 14	0.7027 ± 0.0012
126951.1.2	ETH	hib-26	2942	2854 ± 14	0.7009 ± 0.0012
126951.1.3	ETH	hib-26	2942	2848 ± 14	0.7015 ± 0.0012
126952.1.2	ETH	hib-26	2943	2867 ± 14	0.6999 ± 0.0012
126953.1.2	ETH	hib-26	2944	2840 ± 14	0.7022 ± 0.0012
126954.1.2	ETH	hib-26	2945	2857 ± 14	0.7007 ± 0.0012
126954.1.3	ETH	hib-26	2945	2855 ± 14	0.7009 ± 0.0012
126955.1.2	ETH	hib-26	2946	2850 ± 14	0.7013 ± 0.0012
126956.1.2	ETH	hib-26	2947	2854 ± 14	0.7010 ± 0.0012
127139.1.2	ETH	lfs-9	2351	2356 ± 13	0.7458 ± 0.0012
127140.1.2	ETH	lfs-9	2352	2341 ± 13	0.7472 ± 0.0012
127141.1.2	ETH	lfs-9	2353	2383 ± 13	0.7433 ± 0.0012
127142.1.2	ETH	lfs-9	2354	2408 ± 13	0.7410 ± 0.0012
127143.1.2	ETH	lfs-9	2355	2400 ± 13	0.7417 ± 0.0012
127144.1.2	ETH	lfs-9	2356	2406 ± 13	0.7411 ± 0.0012
127145.1.2	ETH	lfs-9	2357	2414 ± 13	0.7404 ± 0.0012
127146.1.2	ETH	lfs-9	2358	2413 ± 13	0.7405 ± 0.0012
127147.1.2	ETH	lfs-9	2359	2427 ± 13	0.7392 ± 0.0012
127148.1.2	ETH	lfs-9	2360	2421 ± 13	0.7398 ± 0.0012
127149.1.2	ETH	lfs-9	2361	2433 ± 13	0.7387 ± 0.0012
127150.1.2	ETH	lfs-9	2362	2446 ± 13	0.7375 ± 0.0012
127151.1.2	ETH	lfs-9	2363	2424 ± 13	0.7395 ± 0.0012
127152.1.2	ETH	lfs-9	2364	2427 ± 15	0.7392 ± 0.0014
127152.1.3	ETH	lfs-9	2364	2451 ± 13	0.7371 ± 0.0012
127153.1.2	ETH	lfs-9	2365	2463 ± 13	0.7360 ± 0.0012
127154.1.2	ETH	lfs-9	2366	2431 ± 13	0.7389 ± 0.0012
127155.1.2	ETH	lfs-9	2367	2467 ± 15	0.7356 ± 0.0014
127155.1.3	ETH	lfs-9	2367	2437 ± 15	0.7384 ± 0.0014
127156.1.2	ETH	lfs-9	2368	2440 ± 13	0.7380 ± 0.0012
127157.1.2	ETH	lfs-9	2369	2421 ± 13	0.7398 ± 0.0012
127158.1.2	ETH	lfs-9	2370	2429 ± 15	0.7391 ± 0.0014

127158.1.3	ETH	lfs-9	2370	2452 ± 13	0.7369 ± 0.0012
127159.1.2	ETH	lfs-9	2371	2431 ± 13	0.7389 ± 0.0012
127160.1.2	ETH	lfs-9	2372	2420 ± 13	0.7399 ± 0.0012
127161.1.2	ETH	lfs-9	2373	2432 ± 13	0.7388 ± 0.0012
127162.1.2	ETH	lfs-9	2374	2435 ± 15	0.7385 ± 0.0014
127162.1.3	ETH	lfs-9	2374	2457 ± 13	0.7365 ± 0.0012
127163.1.2	ETH	lfs-9	2375	2424 ± 13	0.7395 ± 0.0012
127164.1.2	ETH	lfs-9	2376	2427 ± 13	0.7393 ± 0.0012
127165.1.1	ETH	lfs-9	2377	2435 ± 15	0.7385 ± 0.0014
127166.1.1	ETH	lfs-9	2378	2413 ± 15	0.7405 ± 0.0014
127167.1.1	ETH	lfs-9	2379	2420 ± 15	0.7399 ± 0.0014
127168.1.1	ETH	lfs-9	2380	2432 ± 15	0.7388 ± 0.0014
127169.1.1	ETH	lfs-9	2381	2438 ± 15	0.7383 ± 0.0014
127170.1.1	ETH	lfs-9	2382	2450 ± 15	0.7371 ± 0.0014
127171.1.1	ETH	lfs-9	2383	2443 ± 15	0.7377 ± 0.0014
127202.1.1	ETH	vokb-79	2464	2425 ± 14	0.7394 ± 0.0013
127202.1.2	ETH	vokb-79	2464	2439 ± 14	0.7382 ± 0.0013
127203.1.1	ETH	vokb-79	2465	2432 ± 14	0.7388 ± 0.0013
127204.1.1	ETH	vokb-79	2466	2463 ± 14	0.7360 ± 0.0013
127205.1.1	ETH	vokb-79	2467	2473 ± 14	0.7350 ± 0.0013
127205.1.2	ETH	vokb-79	2467	2459 ± 14	0.7363 ± 0.0013
127206.1.1	ETH	vokb-79	2468	2463 ± 14	0.7360 ± 0.0013
127207.1.1	ETH	vokb-79	2469	2444 ± 14	0.7377 ± 0.0013
127208.1.1	ETH	vokb-79	2470	2458 ± 14	0.7364 ± 0.0013
127208.1.2	ETH	vokb-79	2470	2434 ± 14	0.7386 ± 0.0013
127209.1.1	ETH	vokb-79	2471	2459 ± 14	0.7363 ± 0.0013
127210.1.1	ETH	vokb-79	2472	2452 ± 14	0.7369 ± 0.0013
127211.1.1	ETH	vokb-79	2473	2471 ± 14	0.7352 ± 0.0013
127211.1.2	ETH	vokb-79	2473	2448 ± 14	0.7373 ± 0.0013
127212.1.1	ETH	vokb-79	2474	2489 ± 14	0.7335 ± 0.0013
127213.1.1	ETH	vokb-79	2475	2479 ± 14	0.7345 ± 0.0013
127213.1.2	ETH	vokb-79	2475	2475 ± 14	0.7349 ± 0.0013
127214.1.1	ETH	vokb-79	2476	2443 ± 14	0.7378 ± 0.0013
127215.1.1	ETH	vokb-79	2477	2453 ± 14	0.7369 ± 0.0013
127216.1.1	ETH	vokb-79	2478	2455 ± 14	0.7367 ± 0.0013
127216.1.2	ETH	vokb-79	2478	2436 ± 14	0.7384 ± 0.0013
143523.1.1	ETH	ahmo-63	2948	2863 ± 14	0.7002 ± 0.0012
143524.1.1	ETH	ahmo-63	2949	2842 ± 14	0.7020 ± 0.0012

References

Baillie, M. G. (1982). *Tree-ring dating and archaeology.* London : Croom Helm. Retrieved from http://lib.ugent.be/catalog/rug01:002175788

Baillie, M. G., & Pilcher, J. R. (1973). A simple cross-dating program for tree-ring research. *Tree-Ring Bulletin, 33*, 7-14.

Brown, D. M., Munro, M. A., Baillie, M. G., & Pilcher, J. R. (1986). Dendrochronology—The Absolute Irish Standard. *Radiocarbon, 28*, 279–283. doi:10.1017/S0033822200007372

Eckstein, D., & Bauch, J. (1969). Beitrag zur Rationalisierung eines dendrochronologischen Verfahrens und zur Analyse seiner Aussagesicherheit. *Forstwissenschaftliches Centralblatt, 88*, 230–250. doi:10.1007/BF02741777

Herbst, K., Muscheler, R., & Heber, B. (2017). The new local interstellar spectra and their influence on the production rates of the cosmogenic radionuclides ^{10}Be and ^{14}C. *Journal of Geophysical Research: Space Physics, 122*, 23-34. doi:10.1002/2016ja023207

Hollstein, E. (1980). *Mitteleuropäische Eichenchronologie : Trierer dendrochronologische Forschungen zur Archäologie und Kunstgeschichte.* Mainz am Rhein : von Zabern. Retrieved from http://lib.ugent.be/catalog/rug01:002192863

Munro, M. A. (1984). An Improved Algorithm for Crossdating Tree-Ring Series. Retrieved from http://hdl.handle.net/10150/261251

Nicolussi, K., Kaufmann, M., Melvin, T. M., van der Plicht, J., Schießling, P., & Thurner, A. (2009). A 9111 year long conifer tree-ring chronology for the European Alps: a base for environmental and climatic investigations. *The Holocene, 19,* 909-920. doi:10.1177/0959683609336565

Nicolussi, K., Weber, G., Patzelt, G., & Thurner, A. (2015). A question of time: extension of the Eastern Alpine Conifer Chronology back to 10071 b2k. *GFZ Potsdam, Scientific Technical Report STR15, Potsdam*, 69-73.

Pichler, T., Nicolussi, K., Schröder, J., Stöllner, T., Thomas, P., & Thurner, A. (2018). Tree-ring analyses on Bronze Age mining timber from the Mitterberg Main Lode, Austria - did the miners lack wood? *Journal of Archaeological Science: Reports, 19*, 701-711. doi:https://doi.org/10.1016/j.jasrep.2018.02.039
